# Family migration and well-being of Chinese migrant workers’ children

**DOI:** 10.1038/s41598-024-63589-5

**Published:** 2024-06-04

**Authors:** Ling Tang, Xin Xiang, Yang Liu

**Affiliations:** 1 College of Humanities and Social Sciences, Shenzhen Technology University, Shenzhen, Guangdong China; 2 Faculty of Education and Psychological Science, Yuncheng University, Yuncheng, Shanxi China; 3 Faculty of Political Science and Sociology, Rheinische Friedrich-Wilhelms-University of Bonn, Bonn, Germany; 4 The Marxism Institute at Guangdong Ocean University, Zhanjiang City, China; 5 Economic Faculty, Shenzhen University, Shenzhen, China; 6 Science & Technology Innovation & Service Centre, Guangming District, Shenzhen Municipal Government, Shenzhen, China

**Keywords:** Chinese migrant workers’ children, Social cognitive well-being, Lifelong satisfaction, Vocational education and training school, Psychology, Health care

## Abstract

This article aims to explore the effects of parental migration on the well-being of children and how to adjust social cognitive well-being through the interrelations among family relations and social cognitive well-being indicators using structural equation modelling. Two modified social cognitive well-being models were tested in 1682 Chinese migrant workers' children to examine the pathways among social cognitive well-being and family relation characteristics. The modified models are based on the social cognitive well-being model and the characteristics of Chinese migrant workers' children. The results show that caregiver-child communication frequency, caregiver-child regulation, caregiver-child conflicts, caregiver-child trust and communication, and coactivity positively impact children’s social cognitive well-being. In contrast, caregiver-child alienation negatively influences children’s social cognitive factors through caregiver-child trust and communication. Additionally, this research revealed that family-related characteristics (caregiver-child regulation, caregiver-child coactivities, caregiver-child communication frequency, caregiver-child alienation, caregiver-child conflicts, and caregiver-child trust and communication) are interconnected with social cognitive well-being indicators (academic satisfaction, outcome expectations, goal progress, lifelong satisfaction, environmental support, positive affect, negative affect, and self-efficacy). This suggests that family migration and relationships with caregiver(s) can significantly affect the well-being of migrant workers' children.

## Introduction

At the end of the twentieth century, urbanization and modernization brought millions of Chinese workers from rural to urban areas of China. These people search for a better life and usually have no choice but to migrate. Millions of Chinese migrant workers devote their youth and energy to the construction of modern China. They are considered “the cost of the Chinese economy” because of their limited income and lack of time to take care of their children. Migrant workers either left their children behind in their hometowns in rural areas or brought them with them to the city. The quality of life and social welfare of urban and rural citizens differ, with migrant workers and their children finding it challenging to settle down and adapt. Additionally, because of the long absences of migrants from their farm work in their place of origin, they struggle to make a living when they return to their hometown. This phenomenon has been described as “the price of Chinese economic development”.

In May 2021, the National Bureau of Statistics issued the Seventh National Census Bulletin (No. 7), revealing that in 2020, the number of migrant children in China was approximately 130 million^1^. Among them, there were 71.09 million migrant children within China, while the number of left-behind children, including those in rural areas, totalled 66.93 million. The "2023 Rural Education Development Report" indicates a decrease in the number of left-behind children in rural China to 9.02 million in 2022^2^. This reduction reflects the county urbanization acceleration and improvements in universal education facilities, indicating a partial alleviation of the left-behind children problem in rural areas. However, this topic remains entirely unresolved. In 2024, an analysis of the results of a survey on the mental health status of 515 rural left-behind children revealed that they experienced learning disabilities, moral deficiencies, and behavioural disorders. The mental health of rural left-behind children is significantly lower than that of non-left-behind children, indicating a substantial difference between the two groups. This suggests that rural left-behind children are more susceptible to psychological and behavioural problems^3^. Identifying the factors that influence family migration and the well-being of migrant workers' children could improve the mental health status of these children and might help tackle psychological and behavioural problems.

As well-being research originates in Western culture, which emphasizes individualism, whereas Chinese culture is a typical collectivist culture, the conditions for well-being in individuals are likely to differ significantly. This study considers family relations as one of the components of the social cognitive well-being model. One of the few academic frameworks that illustrates human strengths and positive adjustment is the social cognitive model of well-being^4^. The framework emphasizes the mediating functions of key predictors of positive adjustment, such as self-efficacy, outcome expectation, and goal progress, in mediating the relationship between affective traits/personality (positive or negative affect) and environmental factors (environmental support and obstacles) and well-being outcomes^5^. Six family relationship factors (caregiver-child coactivity, caregiver-child communication frequency, caregiver-child regulation, caregiver-child conflicts, caregiver-child alienation, and caregiver-child trust and communication) were involved in the environmental factors of the social cognitive model of well-being in this study and were divided into two parts. In this research, structural equation modelling was used to assess the pathways of these two new models.

## Literature review

### Mental health studies of migrant workers’ children

Previous research on the children of Chinese migrant workers has focused on demography, education, sociology, and psychology. Most studies are about left-behind children, and some on migrant children. Due to the absence of parental care and parental affection^6^, left-behind children may be more likely to suffer from negative effects, such as anxiety^7^, victimization^8^, depression^9^, loneliness^10^ and emotional trouble^1^, than children from nonmigrant families^11^. Meanwhile, left-behind children of migrant workers in rural areas are encouraged to study agricultural-related majors in VET schools. The government offers an allowance and no tuition fees for rural students enrolled in VET schools^12^.

Additionally, migrant workers’ children have been studied and compared through different identities, which are based on family migration arrangement types. Generally, there are the following categories of family migration (shown in Table 1):

**Table 1:** Family Migration Arrangement Types.

	Live with	Migrant with	Left behind by
Live_with_parents	Both parents	Both parents	Not applicable (Nonmigrants)
Live_with_1_parent	Only Father; Only Mother	Only Father; Only Mother	Only Mother; Only Father
Live_with_others	Grandparent(s); Siblings; Others	Others	Both parents
Live_alone	No one	Migrant alone	Both parents

Zhao et al.^13^ noted that adolescents left behind by both parents have mental difficulties in different aspects, such as emotions, social behaviour, and peer relationships. Jorden and Graham found that children left behind by their mothers appear particularly unsatisfied compared with children in nonmigrant families^14^. Moreover, in the research of Cortes^15^, children left behind by their mothers are less happy and more likely to suffer from detrimental effects than children left behind by their fathers. Graham and Jordan^16^, in a study of left-behind children in Thailand and Indonesia, found that children left behind by their fathers are more likely to be unhappy. Mazzucato et al.^17^ reported that children left behind by both parents seem to have worse psychological health than those left behind by only one parent. Additionally, Chen and Chan^9^ reported that left-behind children living in divorced or single-parent families are at increased risk of depression and negative emotions. In the current well-being literature, left-behind children are more often compared to those from nonmigrant families^14,16–18^. Research comparing left-behind children with migrant young people remains insufficient.

According to a nationwide study of children of Chinese migrant workers, left-behind children frequently experience long-term separation from their parents^19^. Many studies have shown that left-behind children are more prone to accidents (such as animal bites, falls, cuts or piercings, road accidents, and burns or scalds) and crimes (such as abduction, sexual assaults, and theft) than other rural children^20^. Children who are left behind are frequently vulnerable to sexual assault since they lack the perception and ability to protect themselves against molesters^13^.

The majority of children who are separated from their parents experience emotional distress. According to a survey conducted in Hunan, Anhui, and other Chinese provinces, more than 80% of left-behind young people have mental illnesses^21^. Furthermore, according to a Sichuan Province poll, 60% of left-behind children claimed that their caregiver(s) did not treat them or their parents, and a similar number of young people did not want their parents to leave home or work in cities^22^. The relatives, usually grandparents, who care for left-behind children struggle to meet the psychological and emotional demands of developing young people^20^. Another survey revealed that 68% of left-behind children seldom talk to their caregivers^23^. Left-behind children are more likely to experience unpleasant feelings such as loneliness^10^, sadness^9^, being easily angered, intolerance, low self-esteem^20^, and anxiety^7^. Left-behind children are also more likely to have poor academic performance, drop out of school, participate in public disturbances, experience larceny^24^, confront teachers and classmates, and engage in suicidal behaviour^25^.

Lower family income, less parent–child communication, social discrimination, and the limitations of the household registration system (which include marginalization, an unfavourable path to schooling, unfavourable social and medical welfare, and barriers to engagement in urban life) all contribute to migrant youth having more mental health problems and being more likely to be victims of or perpetrators of, crime. Furthermore, unfavourable consequences develop among migrant adolescents as a result of their new and unpredictable living conditions, which frequently induce trauma and may lead to improper actions^25^. Despite major injuries and challenges, migrant workers are increasingly choosing to carry their children with them instead of abandoning them in rural regions^20^. All of these statistics highlight the significance of mental health treatment for both left-behind children and migratory teenagers.

Generally, research on migrant workers’ children has the following characteristics. First, many previous studies have focused on individuals in early childhood^26^, such as students in primary school^10^ and children below junior high school^27^. Although adolescence is crucial to an individual’s development and well-being^28,29^, little research has focused on migrant young people or left-behind adolescents. Second, some existing well-being studies are only concerned with migrant young people^26,30^, while others include only left-behind children^8,11^. Limited well-being studies are concerned with both migrant youngsters and left-behind adolescents regarding their family arrangements and relations. Even where the research included both^31^, the study was conducted from the parents’ perspective. Third, even in cases where some research has focused on migrant children, studies have concentrated on children’s mental^29^ and physical health^32^ in schools. Additionally, migrant children may be affected by negative emotions because of family migration^33^. The term “migrant workers' children” in this study refers to adolescents who were born to families with migrant parents or parents.

### Social cognitive well-being model (SCWB)

**Figure 1:**
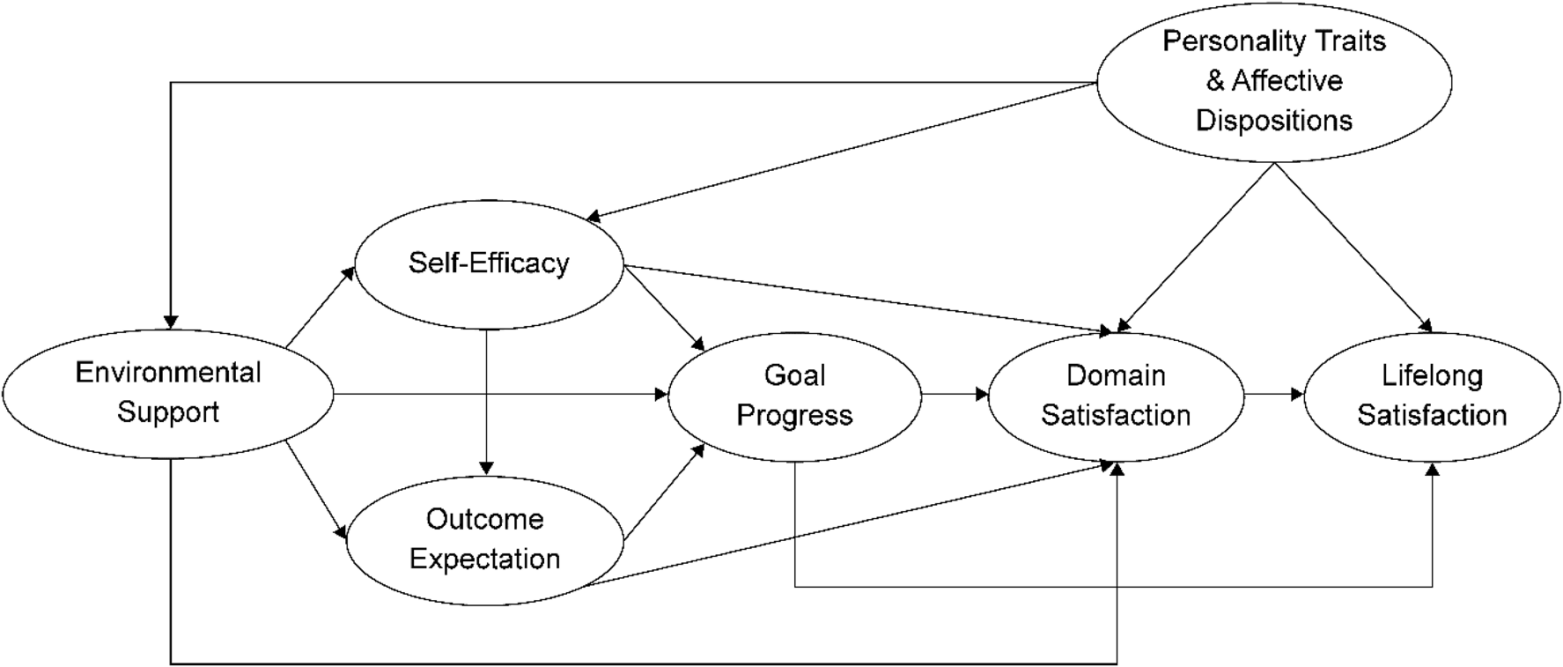
Social Cognitive Well-being Model. This figure is based on Lent’s^4^ description of the social cognitive well-being model.

Theoretically, the social cognitive model of well-being (SCWB) originated from social cognitive career theory (SCCT)^34^. SCCT is based on Bandura's general social cognitive theory^35^. The SCCT encompasses four interrelated models of academic/career interest development, choice, performance, and academic/career satisfaction^34^. SCWB theory modifies SCCT by adding self-efficacy^36^, learning progress, outcome expectations, and environmental support to predict academic satisfaction^37^. The SCWB model creates a framework among seven elements (Fig. 1)^4^. Additionally, the paths and relationships among these seven elements were determined (Fig. 1). The SCWB model focuses on the joint participation of environmental, behavioural, cognitive, and affective factors that increase people’s satisfaction^4^ in a particular domain (especially in education and careers) and throughout life.

### Chinese perspective on well-being

Since well-being research originated in Western culture, which emphasizes individualism, while China is a typical collectivist culture of the East, the conditions for well-being in individuals could differ significantly. In Chinese culture, there are reflections of well-being descriptions from Confucianism, Taoism, and Buddhism (the three most influential philosophical concepts in China).

According to Confucianism, well-being is not only the well-being ideal of the moral elite but also the pursuit of the public, covering both internal dimensions and an external level of well-being. It runs through the confrontation and integration between moral well-being and utilitarian happiness^38^.

Comparatively, Taoism has shifted from focusing on the level of people in social strata to pursuing the idyllic existence of people on the natural scale. For Taoism, well-being is not a kind of enjoyment but the epiphany and transcendence of mind. Laozi thinks that all the goods are complicated, and the things you get are not as good as your body and mind^39^.

Buddhism believes that life has no absolute eternal happiness, all living beings’ lives are suffering, and only when it reaches “Nirvana” can it be genuinely detached. Physical and mental practices, including meditation, giving, and eliminating all desires, can help the soul of individuals enter the realm of Nirvana and achieve happiness or well-being. In general, the well-being of Buddhism is being clear-minded, pursuing the original self and harmony with society^40^.

Overall, based on Eastern philosophies, the Chinese view of well-being emphasizes people’s roles and obligations in society and combines individuals’ inner worlds with their surroundings and family obligations^41^. Moreover, instead of personality, Chinese people value harmonious societal relations and view well-being as seeking a balance between the inner world and social relationships^40^. According to Gao et al.^39^, the Chinese view of well-being starts from a socially oriented self, while in Western culture, it starts from an individual-oriented person.

### Comments and limitations of previous well-being studies

Extant well-being studies can be classified into several categories based on different dimensions of well-being. According to Lent^4^, there are three main well-being dimensions: temporal, contextual, and cultural.

First, temporal dimensions include global lifelong well-being, which is context-free. This is followed by intermediate-term well-being, which lasts for a certain period (school, career, or home life), and immediate “online” well-being, which is the current role of an individual (student, worker, or family member). The cultural dimensions of well-being mainly include the universalist, individualistic, and collectivist dimensions^4^. Western culture is defined as individualism, while Eastern culture is defined as collectivism^4,42^.

The definition of well-being in the social psychological literature stems from Western culture. Most previous studies^43^ have focused on an individual’s feelings (SWB), purpose (PWB), or both (SCWB). Even though the SCWB mentions environmental support, it seems very general. While it may work well in Western culture, in Eastern cultures such as China, people’s well-being is largely connected to relations in certain groups^4^. Therefore, no current research models (SWB, PWB, and SCWB) can correctly explain the well-being condition in a collectivist culture due to a lack of detailed information on their group relations and surroundings.

Regarding the contextual dimension, this study focuses on the well-being of migrant workers’ children. Most of the existing well-being literature on migrant workers’ children focuses on preschool children or children in early childhood^9,27,43^. Related studies for adolescents still need more information, which contradicts the reality that mental health disorders and psychological problems are pervasive in adolescence^44^. Moreover, there are limited well-being research studies concerned with migrant youngster classifications (migrants with both parents, migrants with only mothers, and migrants with only fathers) and comparisons among them. Therefore, this study compares the differences among those adolescents: a. left behind by parents, grandparents, or other caregivers as primary caregivers; b. only the father migrates (left behind by father); c. only the mother migrates (left behind by mother); d. migrates with both parents; e. migrant with only one parent; f. nonmigrant family in rural areas; and g. nonmigrant families in urban areas.

The previous well-being literature and its limitations offer several directions for this research study. First, relations and surrounding factors should be added to the current well-being models for research participants from Eastern cultures. Second, previous findings require additional verification with data from other domains of students’ lives (such as social, family, and financial)^42^. Compared with others, family relations could be essential to the well-being of migrant workers’ children. In contrast, systematic family-related well-being research on migrant workers’ children still lacks evidence. Third, it is essential to extend the study to more diverse participants of different ages and life contexts (such as adolescents in high school, employees, and retired workers)^45^. Fourth, it would be useful to tell insightful stories of those factors that carry in-depth knowledge of family relations and well-being. Mixed methods that combine qualitative and quantitative studies are also essential for expanding current studies^5^. Fifth, although several studies have investigated self-construal, few have investigated Chinese migrant workers’ children or VET school students. Moreover, despite a consultancy centre providing certain consulting services to students in VET schools in China, especially in urban areas, limited VET schools are concerned with mental health consultation for migrant young people. In some rural areas, local governments have set up centres for left-behind children to provide consulting services; however, they are normally not professional, lack scientific consulting tools, and mostly concentrate on left-behind children in their early childhood instead of adolescents.

### The modified social cognitive well-being model based on Chinese VET school students' characteristics

A new model has been developed based on previous well-being studies (primarily the normative well-being model: SCWB) and the characteristics of migrant workers’ children.

**Figure 2:**
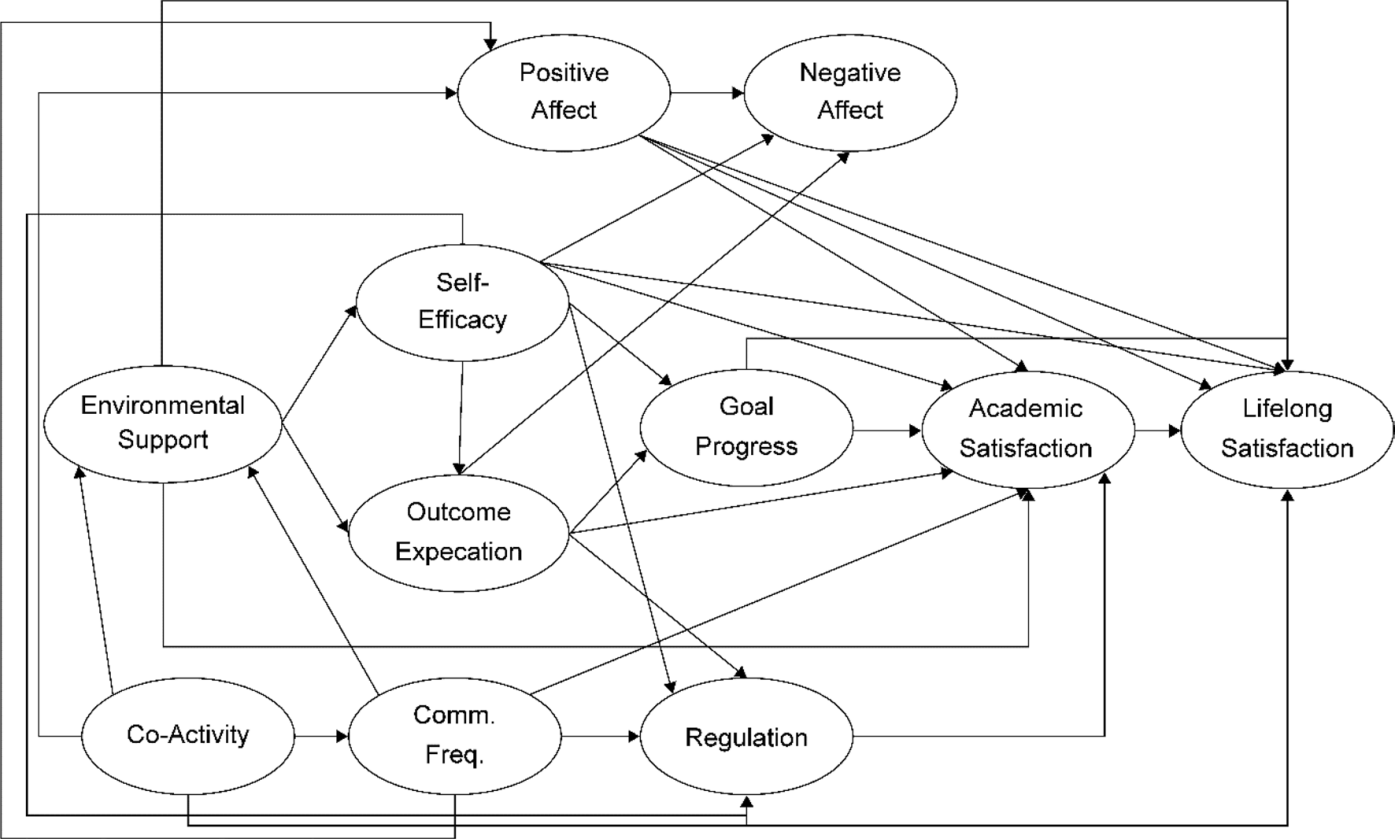
Family-Related Modified Social Cognitive Model I. Comm. Freq. is short for caregiver-child communication frequency in this figure.

**Figure 3:**
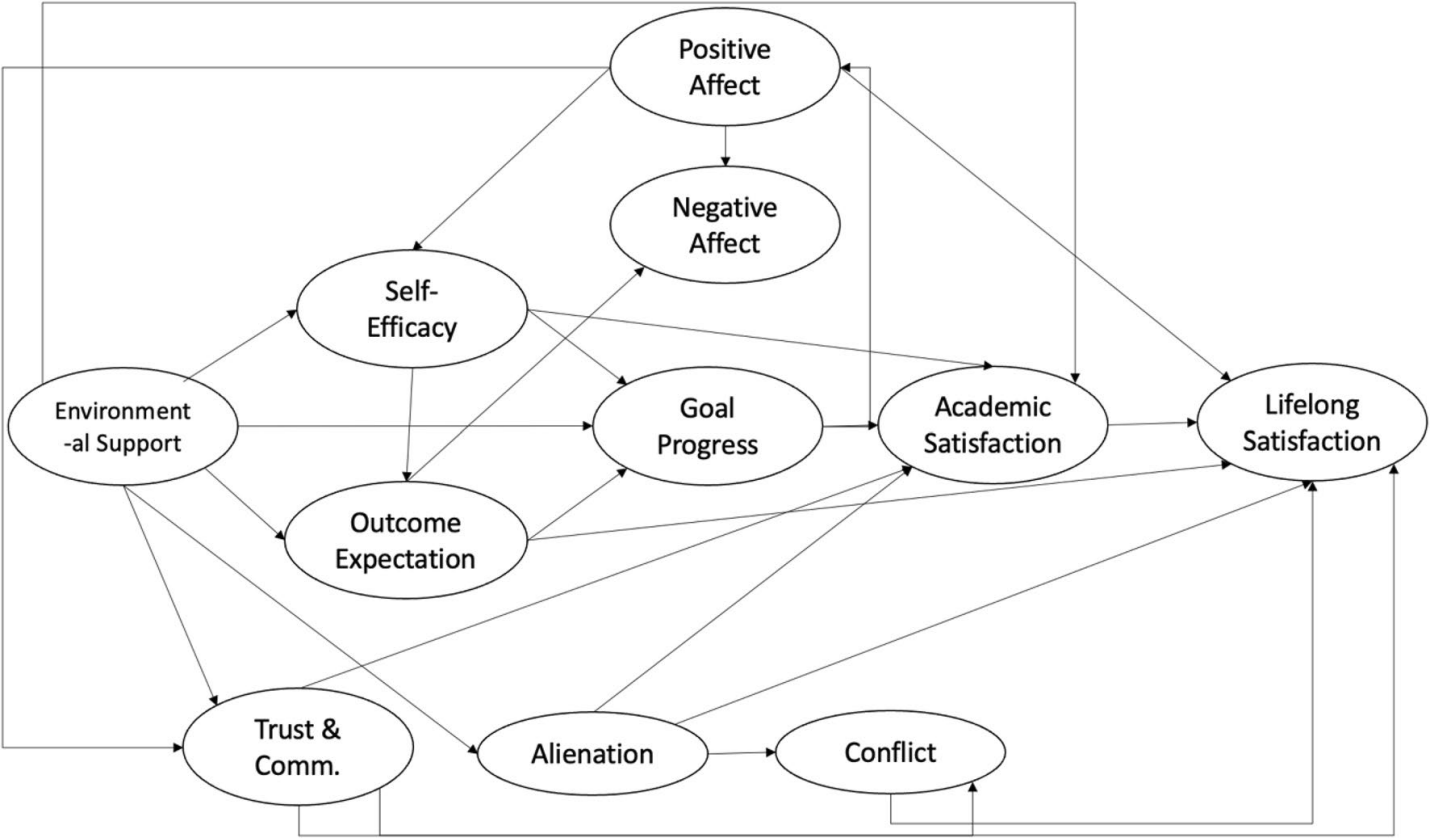
Family Relationships and Social Cognitive Model II. Trust & comm. is short for caregiver-child trust & communication, Alienation is short for caregiver-child alienation, and conflict is short for caregiver-child conflict in this figure.

Figures 2 and 3 are based on previous studies^4,46–49^ and describe the Collectivism SCWB models. In an Eastern cultural context, people value harmonious societal relations and view well-being as seeking a balance between an individual’s inner world^49^ and social relationships^40^. The environmental support indicator in the previous SCWB model is general; therefore, the new model includes family relationships based on Eastern cultural characteristics. Additionally, the previous trial model testing stage included the participants’ view of social relations in the cultural dimension: self-construal factors (interdependence and independence). However, self-construal factors do not have statistical significance and complicate the model; thus, this study excluded self-construal factors.

The family environment could differ for children due to their parents’ migration; thus, several indicators were added to the SCWB. These are described below (Figs. 2 and 3).

#### Caregiver–child attachment

Attachment is a lasting emotional bond with persisting strength. It includes three parts: trust, communication, and alienation. The basic matter of attachment theory is the influence of optimal and nonoptimal social attachment on psychological adaptation. For a long time, the relationship between a family’s relationships and a person’s character and well-being has interested developmental psychologists. Parent–child attachments are significantly related to mental health^50^. The caregivers of the children of migrant workers could be parents or others; therefore, the parent–child attachment is replaced by the caregiver-child attachment.

#### Caregiver-child coactivities

Compared to others, children from migrant workers’ families might suffer from the absence of parental care. In the case of left-behind children, their parents left them with other caregivers (normally old grandparents); migrant youngsters, their parents are busy with their work and struggle to survive in the migrant city with limited time to get together with their children^51^. Therefore, both of these groups lack caregiver-child coactivity. This study aimed to compare caregiver-child coactivity between children of migrant workers and those of nonmigrant families.

#### Caregiver–child communication frequency

The frequency and quality of communication between parents/caregivers and children are related to adolescents’ mental health, especially for migrant workers’ children^52^. In later childhood, children normally receive more support and communication, especially from their mothers, while in adolescents, it seems that parents tend to reduce mental control and increase tolerance. Family communication and support also decreased. This may reflect parental response to children’s requests for greater independence and impact their well-being^53^.

#### Caregiver–child regulation

Parental/caregiver(s) regulation rules can predict children’s academic achievement. Moreover, it mediates the negative association between parental absence and children’s school performance. Lower levels of parental regulation, parent–child communication, and parent–child coactivities were correlated with greater depression^52^.

#### Caregiver–child conflicts

Conflicts between parents and adolescents are highly correlated with school misconduct and antisocial and at-risk behaviour. Conflicts within migrating families could be caused by generation gaps and changeable living environments^54^. These family conflicts are identified as a form of adaptive pressure in a specific period^55^ and other areas that adjust to different stresses, for instance, physical, economic, and social.

Lent^4^ noted that the variables that influence an individual’s well-being can be divided into three main categories. This new model is also based on these three categories.

- The first category relates to biological variables, such as personality, affect, and emotion; these are commonly considered to be genetic factors based on biological mechanisms^56^, which may be useful predictors that influence SWB^4^. Other psychologists have argued that cognitive and motivational factors might mediate the relationship between biological variables and well-being^57^. Although personality traits and affective dispositions are somehow determined by genetic traits, they are less stable over longer time intervals^58^. Positive affect is reported as a component of SWB and shares the family environment’s estimate with it, which suggests that it is also a factor related to experiential variables, such as family and social surroundings^59^. Moreover, situational factors may also impact short-term emotions or enjoyment of life^36^. Thus, biological variables may be measured, intervened in, or adjusted through related mediating variables. In the new model, personal traits and affective dispositions were removed, and a modified social-cognitive model was created.
- The second category relates to social, cognitive, and behavioural variables. Compared with innate traits and qualities, these variables are more likely to be modifiable constructs amenable to self-control. On the one hand, social, cognitive, and behavioural variables are more flexible and may intervene in promoting well-being. On the other hand, they also have close interactions with biological variables; they potentially mediate and moderate the relationships between the latter and well-being^1^. Three types of cognition have been studied in several empirical studies: (i) self-efficacy, which originates from personal control beliefs; (ii) outcome expectations, which are about people’s beliefs about their future life^60^; and (iii) goal progress, which refers to people’s determination to attain a certain level of performance or outcome^4,36^. Lent^4^ noted that personal beliefs may partly mediate the relationship between goal progress and outcome expectations. According to Ryan and Deci^61^, people’s goals or values are essential factors that impact SWB. Moreover, they also argue that compared to people with internal goals, people with external or material-oriented goals have lower satisfaction. Additionally, they feel that confidence in achieving valued goals helps promote well-being.
- The third category of well-being-related variables is behaviour and social variables, which emphasize the belief that people achieve some goals through involving themselves in some activities that contribute to personal well-being^61^. This behavioural involvement helps people progress in their goals (e.g., self-set, culturally valued) and brings satisfaction by bringing people into mutual social contacts^62^. Environmental support^63^ and relational resources^64^ have been mentioned as relating to well-being enhancement in many research studies^4^. Some evidence in previous studies shows several benefits of social support, including material/economic support, companionship, and emotional help^65^. In the modified model, environmental and social support are divided into three dimensions (based on the collectivist culture): i. Family relations, which mainly include the quality of parent-child relationships and support from family. ii. School relations include teacher-student relations and support and peer relations. iii. Social support is a variable from a societal perspective. It includes economic elements, social status, financial support, and social identity. In this research, family relationships and the SCWB were studied.

### Migrant workers’ children’s studies

In 2018, there were up to 288.36 million internal migrants in China^66^, representing the largest rural–urban migration in human history considering its sheer size and rate^67^. Due to the trend in migration over the last 30 years, there were 69.7 million left-behind children in 2016^68^, and more than 13.67 million children migrated with their parents^30^.

According to a national survey of Chinese migrant workers’ children, left-behind children often undergo long-term separation from their parents^20^. Many studies have revealed that left-behind children are more vulnerable to accidents (e.g., animal bites, falls, cuts or piercing, traffic accidents, and burns or scalds) and crimes (e.g., abduction, sexual offences, and theft) than other rural children^21^. Left-behind children are usually defenceless against sexual violence because they lack the perception and capacity to defend themselves from molesters^14^. Separation from parents produces a form of mental distress for the majority of left-behind children. According to a survey of Hunan, Anhui, and several other provinces in China, more than 80% of left-behind children have mental health problems^22^. Moreover, according to a survey in Sichuan Province, 60% of left-behind children reported that their caregiver(s) did not treat them or their parents, and the same number of children did not want their parents to leave home or work in cities^23^. The relatives, normally grandparents, who care for left-behind children have difficulties fulfilling the psychological and emotional needs of developing children^21^. Another survey revealed that 68% of left-behind children seldom talk to their caregivers^69^.

A study by Ling^26^ revealed that even when migrant youth live and study in urban areas (or are even born there) with their parents, their urban household registration (“chengshi hukou”) status can cause problems. The Compulsory Education Law of the PRC (revised June 29th of 2006) dictates that “Nine years of compulsory education should be provided to all children regardless of gender, race, religion and wealth under an equal environment” (emphasis added). However, “equal environment” is not precisely defined and governments in different cities have various interpretations. Migrant workers normally pay additional school fees to enroll their children in local schools. Additionally, the educational system in China is highly competitive and test-oriented. To be among the prestigious schools and acquire higher fees and donations, schools strive to maintain high academic standards. The children of migrant workers are usually regarded as academically inferior and allocated to ordinary or low-quality schools^70^. To support the education of their children and manage their family expenses, migrant parents normally need to work overtime because they have less time and communication with their children than do local families.

Lower family income, less parent–child communication, social discrimination, and the limitations of the household registration system (which include marginalization, an unfavourable path to schooling, unfavourable social and medical welfare, and barriers preventing engagement in urban life) cause a greater degree of mental health problems in migrant youth and a greater probability of them being victims of, or of committing crime. Moreover, negative outcomes emerge in migrant youth because of their new and unstable living environments, often causing trauma and possibly leading to inappropriate behaviours^71^. Despite enormous unfairness and hardship, migrant workers increasingly choose to bring their children with them instead of leaving them behind in rural areas^21^. These findings reveal the importance of mental health care for both left-behind children and migrant youth.

## Methods

The survey was conducted through stratified sampling according to the areas where migrant workers’ children are located. It includes both migrant young people in inbound (mostly urban) areas and left-behind children in outbound (mostly rural) areas. At the beginning of the survey, all the participants were informed that "the researcher will use anonymized responses for research and publicity purposes. If you agree with your anonymous responses to be used, please answer the questions." The parents or legal guardians of children under 16 were informed.

### Participants

**Figure 4:**
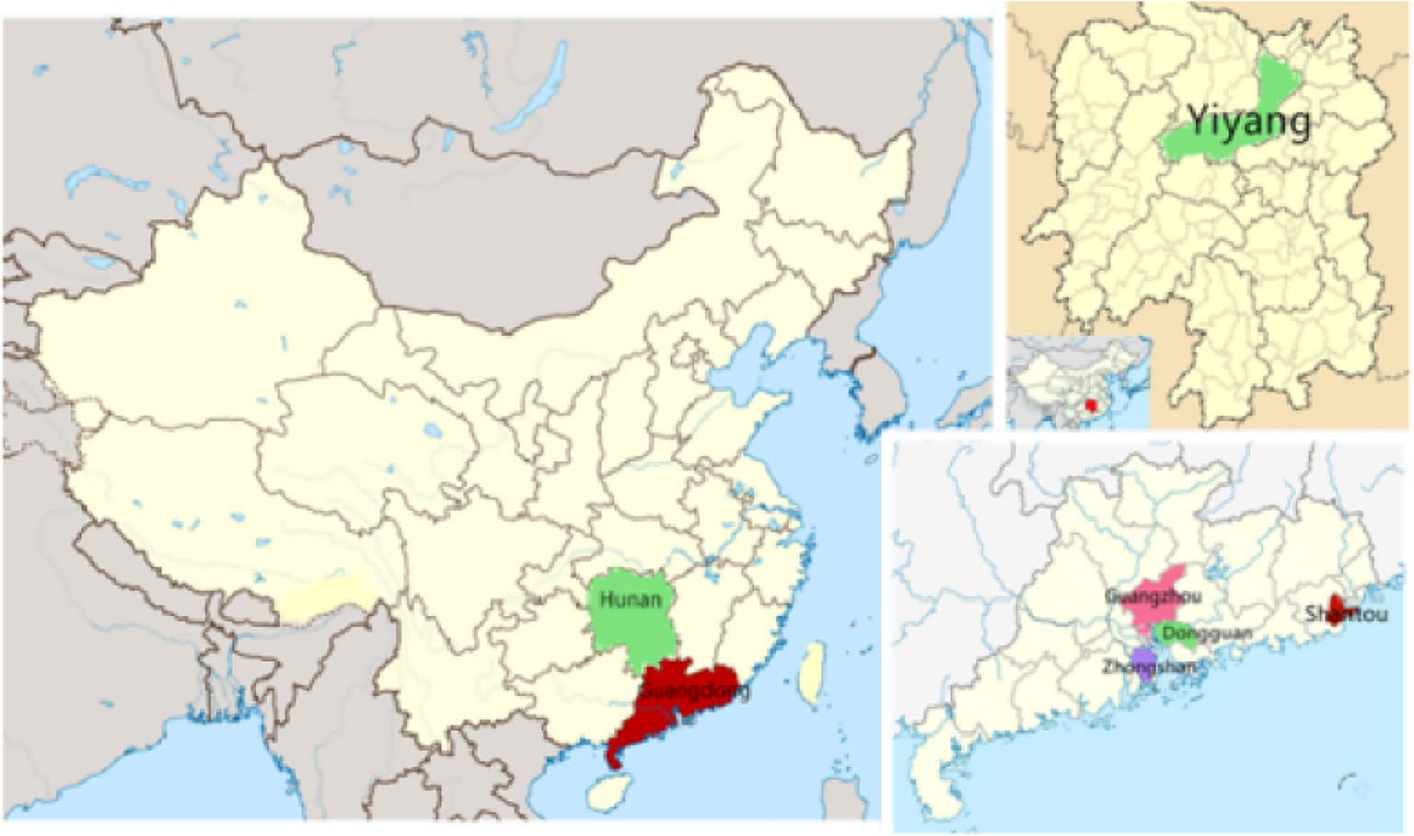
Map of Areas and Cities Involved in this Research. Source: Self-made map based on resources from the Wikimedia Foundation (www.wikimedia.org; The spatial maps were generated from blank maps of China.svg, https://commons.wikimedia.org/wiki/File:China_blank_map_grey.svg; the China Guangdong location map.svg, https://en.m.wikipedia.org/wiki/File:China_Guangdong_location_map.svg; and the location map China Hunan EN.svg, https://zh.wikipedia.org/wiki/File:Location_map_China_Hunan_EN.svg) and edited with Meitu (https://xiuxiu.meitu.com/).

Stratified samples of migrant workers’ children were chosen from 13 VET schools in both Hunan (mainly inbound area) and Guangdong (mainly outbound area) Provinces (shown in Fig. 4). The survey included migrant workers’ children in VET schools from both migrants’ destination and origin areas, specifically, participants located in the Pearl River Delta (inbound area) and Yiyang together with Shantou (outbound area). The VET schools are located in five cities of Guangdong Province (migrants’ destination) and in Yiyang of Hunan Province (migrants’ origin), which have very different economic conditions.

As one of the most popular urbanized areas, the Pearl River Delta in Guangdong Province was chosen for this study. It is one of three areas where most migrant workers gather in China, with approximately 50.72 million migrant workers, approximately 64% of the total number of migrants in Guangdong Province^66^.

Regarding the place of origin of migrant workers, 1.7764 million migrants originated from Hunan Province in 2017, approximately 20% of the central part of China in the same year^66^. Shantou is in southeastern Guangdong Province. It is a city with both inbound and outbound population movement before 2017. The situation changed after 2017, with the outbound rate rising. The net outbound population reached 31.4 thousand^66^.

### Information on the chosen cities

Guangzhou is the capital city of Guangdong Province, with a GDP of $341 billion USD in 2018. Five vocational high schools in different districts were included: the Guangzhou Electronics and Information School, the Guangzhou Trade and Information High School, the Guangzhou City Construction High School, the Guangzhou Trading and Foreign Language School and the Guangzhou Medicine School.

Dongguan is a large city in the Pearl River Delta with a GDP of $120 billion USD in 2018. Two types of vocational high schools in this city have been included: Dongguan Textile & Fashion School and Dongguan Tangxia Polytechnic School.

Zhongshan is a medium-sized city in the Pearl River Delta with a GDP of approximately $55 billion USD in 2018. Two schools were included in this field research: Zhongshan No. 1 Vocational High School and Zhongshan Tanzhou Polytechnic School.

Shantou samples are also included in this research. Shantou is a small city in Guangdong Province, with a GDP of $36.9 billion USD in 2018. One representative school was chosen: Shantou Chaoyang Vocational High School.

Yiyang is a developing town in northern Hunan Province with $25.8 billion USD in GDP in 2018. Not only was the town area included but also the rural and mountainous areas in Yiyang in this field research. Three schools were included: the Nanxian Vocational High School, the Anhua No. 2 Vocational High School and the Anhua No. 1 Vocational High School.

Table 2 shows the data on population, GDP, and area in 2019 for cities included in the stratified sample. Guangzhou, as the capital city of Guangdong Province with an area of 7436 $${km}^{2}$$, is the city with the highest population and GDP ($342 billion USD) among these five cities in 2019; five vocational schools were chosen in Guangzhou, which included 700 interviewed students. Dongguan has one-third of the area (2512 $${km}^{2}$$) and half the population (most of which are migrant workers) of Guangzhou, and its GDP was $137 billion US dollars in 2019. Two vocational schools including 310 students from Dongguan were included. Zhongshan is one of the second-tier cities in the Pearl River Delta megalopolis, with the smallest area (1770 $${km}^{2}$$) and population (3.3 million) among the five chosen cities but with a moderate GDP ($44.9 billion USD). Two schools with 400 students from Zhongshan were included. Yiyang and Shantou were selected as the outbound areas in this research. Compared to Zhongshan, Shantou has a larger area (2123 $${km}^{2}$$) and a greater population (5.6 million) but a lower GDP ($39 billion USD). One vocational school and 190 students were included from Shantou. Among these five cities, Yiyang is the city with the largest area (12,320 $${km}^{2}$$) (constituted mostly of mountains), smaller population (4.4 million) and the smallest GDP ($25.9 billion USD). Three VET schools with 500 students from Yiyang were included^72^.

**Table 2:** Social and Economic Conditions of the Stratified Sample Areas (2019).

Region	City	Population (millions)	GDP (billion USD)	Area ($${\text{km}}^{2}$$)	Schools	n
Inbound	Guangzhou	15.3	$ 342.0	7436	5	700
	Dongguan	8.4	$ 137.0	2512	2	310
	Zhongshan	3.3	$ 44.9	1770	2	400
Outbound	Shantou	5.6	$ 39.0	2123	1	190
	*Yiyang*	4.4	$ 25.9	1320	3	500

Source: National Bureau of Statistics of People’s Republic of China (2019), n = Sample size^72^.

### Pilot studies

With the example of similar studies, the researcher has an idea of the proper sample size for the pretest. A small-scale pilot study is recommended before a large-scale survey can be conducted. Sixty-three university students were included in the trial by Lent et al.^45^. The pretest of this study included 100 students from inbound and outbound areas, among whom 50 were from a vocational school in a rural area in Hunan Province and the other half from a vocational school in Guangdong Province (urban area). According to the feedback from the pilot test, the questionnaire was revised.

The rural areas in Yiyang and the cities of the Pearl River Delta were included in the sample. For the pretest sample, four cities in the inbound area were taken as the migrant workers’ destinations, and three counties in the outbound area of Hunan Province were chosen, all according to the economic conditions of the location.

### Sampling report

The total number of participants in this study was 2,100, which included 1,410 students in urban areas and 690 in rural areas. Thirteen vocational schools are included: four science schools, one medical school, six art schools, and two comprehensive schools. The details are shown in Table 3.

**Table 3:** Schools included in Samples.

	Location	School name	Type	Sample
Inbound	Guangzhou	Guangzhou Electronic and Information School	Science	150
		Guangzhou Trade and Information High School	Arts	150
		Guangzhou City Construction High School	Science	100
		Guangzhou Trading and Foreign Language School	Arts	150
		Guangzhou Medicine School	Medical	150
	Dongguan	Dongguan Textile and Fashion School	Arts	200
		Dongguan Tangxia Polytechnic School	Science	110
	Zhongshan	Zhongshan No.1 Vocational High School	Arts	200
		Zhongshan Tanzhou Polytechnic School	Science	200
Outbound	Shantou	Shantou Chaoyang Vocational High School	Arts	190
	Yiyang	Nanxian Vocational High School	Arts	200
		Anhua No.1 Vocational High School	Comprehensive	200
		Anhua No.2 Vocational High School	Comprehensive	100
Total				2100
No reply				183
No reply of > 15% of survey				235
Effective sample size				1682

Previous studies have shown that incentive measures improve the effectiveness of well-being survey data^5,46,73^. To increase the effectiveness of the data, the headmasters of the 13 VET schools were contacted. The headmasters informed each class teacher about the research and its purpose and asked the students to be sincere when answering the questionnaires in class (the average time to complete the questionnaire was 40 min).

When the 2,100 questionnaires were collected, there were 183 complete nonresponses, 235 of which had more than 15% of the items answered. Using questionnaires with more than 15% missing data could significantly impact any study^74^. Kalton^75^ explained that several methods have been proposed to handle missing data, with the most commonly used solution being the deletion of instances containing at least one missing value for each variable. Given the large size of this study, both the complete nonresponse sample (183) and responses with more than 15% unanswered items (235) were excluded, resulting in an effective sample size of 1682. Although some of the remaining samples still contained missing data, the difference was not statistically significant.

Table 4 shows the information on the participants’ migration family arrangements. To identify the structure of the sample. The family arrangement was identified through five questions:

1. Which one applies best to you?
  A. I am a local
  B. I am not a local, but I come from the same province
  C. I come from another province
2. With whom do you live now?
  A. Parents
  B. Only father
  C. Only mothers
  D. Grandparents
  E. Other
3. “How often do you see your mother?”
  A. At most once a year or never
  B. At least twice a year
  C. At least once a month
  D. At least once a week
  E. Almost every day
4. “How often do you see your father?”
  A. At most once a year or never
  B. At least twice a year
  C. At least once a month
  D. At least once a week
  E. Almost every day
5. What is your parents’ marital status?
  A. Nondivorce
  B. Divorce

**Table 4:** Migration Family Arrangement in Sampling.

Parental presence	N	Migrated with:	n	Left behind by:	n
Live_with_parents	1,063	Both parents	457	/(Nonmigrants)	606
Live_with_one_parent	307	Father Mother	25 52	Mother Father	66 164
Live_with_others	311	Others	0	Both parents	311
Live_alone	1	Lone migrant	0	Both parents	1

Questions 3 and 4 were included to identify the participants who lived in school dormitories and might have chosen *‘other’* in Question 2. Question 5 was added if some participants lived with only one parent because of divorce or death.

The percentage of migrant and left-behind young people has been more than 50% for both inbound (most are urban areas) and outbound (most are rural areas) areas. Regarding the family arrangement for migrant families, the migrant young people in inbound areas can migrate with their mother, father or both parents; left-behind young people in outbound areas could be left behind by their mother, father or both. Most migrant young people migrate with both of their parents (457); more migrate with their mother (52) than with their father (25). Similarly, among young people left behind, more are left by their father (164) than by their mother (66). Within this sample, it is more common for young people to live with their mothers than fathers. There are also migrants in the outbound area (18) and left-behind young people in the inbound area (39) in this sampling, but these are few cases compared to the opposite.

### Measures

In this section, measures and scales that are needed in future studies by researchers are introduced. The scale results were summed and divided by the total number of items in each group. Generally, a higher score implies more positive cognition^48^.

#### Lifelong satisfaction

Diener et al.^76^ produced the 5-item satisfaction subjective well-being scale (SWB). This scale is also called the Satisfaction with Life Scale (SWLS). It was adopted to assess VET students’ lifelong satisfaction. One example question used in this study is “I'm satisfied with my life.” Responses are rated from 1 (strongly disagree) to 5 (strongly agree). Higher scores indicate greater satisfaction. The SWB has generally been shown to have suitable validity and reliability^42,45,77^; in this study, Cronbach’s alpha = 0.805 (Supplemental Data).

#### Academic self-efficacy

Two subscales^28^ were used to measure the self-efficacy of VET students. The first scale was the 5-item learning milestone self-efficacy scale. It is used to assess the confidence of students’ self-regulation abilities in whether they can insist on self-improvement even without supervision. One example of a question used is “how confident will you be in enrolling yourself in your study in the next school year?”^45^. Items are rated from 1 (no confidence at all) to 5 (complete confidence). The other scale was a 7-item coping self-efficacy scale, which measures how confident VET students are in their competence when coping with difficulties or obstacles. One example of a question is “how confident you are in completing your study even under financial pressure.” Responses are rated from 1 (no confidence at all) to 5 (complete confidence). The internal consistency values (Cronbach’s α) in previous studies were between 0.810 and 0.910^5,42,45^. In this study, Cronbach’s α = 0.727 (Supplemental Data), which shows good consistency for this scale.

#### Academic satisfaction

Academic satisfaction was assessed by a 7-item academic satisfaction scale^37^. One item is “Generally, I am satisfied with my academic life.” The responses are rated from 1 (strongly disagree) to 5 (strongly agree), and the higher the score is, the greater the academic satisfaction of the VET students. The reliability estimates ranged from 0.860 to 0.920, and the scores were correlated with other variables in the domain well-being model according to prior studies^5,42,45^. In this study, the Cronbach’s α was 0.884 (Supplemental Data).

#### Academic goal progress

Academic goal progress was assessed by Lent et al.’s^45^ 7-item goal progress scale. We used a modified version with four items to assess the progress of Chinese migrant workers’ children in VET schools (e.g., “how much progress are you making in completing all tasks in every course?”). Items are rated from 1 (no progress) to 5 (excellent progress). Higher scores reflect more considerable progress in the VET student’s academic life. The Cronbach’s alpha coefficients of the scale were 0.87^78^ for Chinese and Turkish students^5^. In this study, it was 0.886 (Supplemental Data).

#### Academic outcome expectations

A 10-item academic outcome expectation scale was adopted to assess the outcome expectations of the VET students. A sample item is “To graduate from a vocational school might help me find a good job.” Items were measured on a 5-point scale from 1 (strongly disagree) to 5 (strongly agree). Higher scores indicate more favourable academic outcome expectations. Sheu et al.^38^ reported a Cronbach’s alpha of 0.94 in a survey of college students in south-central China. Moreover, Cronbach’s alpha was 0.91 in a sample of college students in Italy^79^. In this study, Cronbach’s alpha = 0.902 (Supplemental Data).

#### Environmental support

Environmental support refers to support or resources from teachers, schools, and classmates. According to Lent and Brown^48^, favourable environmental support can promote progress towards one’s goal and improve one’s satisfaction, for example, when students receive strong support from teachers/schools/classmates/parents. Emotional support helps individuals achieve their target more efficiently and feel happier than without such support. Conversely, environmental barriers may prevent someone from achieving their goals and reduce satisfaction. When students confront significant obstacles from teachers or schools, they may become unhappy. Several research studies have shown that environmental support/barriers affect students’ well-being^5^. Moreover, there are different explanations for environmental support. For example, Duffy and Lent^80^ used work conditions instead of environmental support in a modified SCCT well-being model. In this study, work conditions were measured by personal environment and perceived organizational support. The Cronbach’s alpha in this study was 0.747, indicating sufficient reliability (Supplemental Data).

#### Positive and negative affections

We used the Chinese version of the Positive and Negative Affect Scale^81^. It contains two sections: five questions about positive affect (e.g., enthusiastic, proud, excited) and seven questions about negative affect (e.g., lonely, irritable, and nervous). The answers are scored from 1 (never) to 5 (always), and the scores indicate how often the participants have positive and negative feelings in general. The positive and negative subscales showed adequate internal consistency in this study and yielded Cronbach’s alphas of 0.747 for the positive affect subscale and 0.852 for the negative affect subscale (Supplemental Data).

#### Caregiver-child attachment

Before completing this scale, a question with options for caregivers must be given (A. both parents, B. only father, C. only mother, D. grandparents, E. Other__). Based on attachment theory, Armsden and Greenberg^50^ designed the Inventory of Parental Attachment Scale (IPAS) to study parent–child relations. Here, the IPAS assessed the relationships between migrant workers’ children and their caregivers. The IPAS has three dimensions (trust, communication, and alienation), including positive and negative aspects. The Trust Scale contains 10 items (e.g., “my parents/caregivers trust my judgement”), the Communication Scale also includes 10 items (e.g., “my parents/caregivers encourage me to talk about my difficulties”), and the Alienation Scale has eight items (e.g., “I get upset a lot more than my parents know”). In this research, the scales were simplified, and the trust and communication scales were combined; therefore, there were two main scales: the Trust and Communication Scale (nine items) and the Alienation Scale (eight items). Items were measured from 1 (never) to 5 (always). In this research, Cronbach’s alpha = 0.862 for the Trust and Communication Scale, and Cronbach’s alpha = 0.745 for the Alienation Scale.

#### Caregiver‒child communication frequency

This is a 5-item scale; the original scale has five questions related to the mother and five to the father. In this study, they were combined into five questions for caregivers. It was used to test the frequency of parent–child communications. It includes five conditions—school, friends, feelings, teachers, and worries—and is answered with a three-point key (A. never; B. sometimes; C. often^81^) with a Cronbach’s alpha = 0.881 in this study (Supplemental Data).

#### Caregiver-child co-activities

There are five items to assess the frequency of parent–child coactivities, including having dinner, travelling, shopping, watching TV/movies/shows, and doing sports. Items are measured from 1 (never) to 5 (more than once a week). Higher scores indicate a greater frequency of parent–child coactivities. Cronbach’s alpha = 0.73 in the research of^81^. In this study, the item “how often do your parents watch TV with you?” was combined with “how often do your parents watch films/shows with you?” and “how often do parents go shopping with you?” has been added. Cronbach’s alpha = 0.790 in this research (Supplemental Data).

#### Caregiver–child regulation/house rules

We used an 8-item scale^52^ that contains factors from the following three perspectives: socialization (e.g., “Do your caregiver [s] have strict rules on making friends”), academic performance, and screen time. Items are rated from “not at all” to “a great deal.” The alpha coefficient = 0.822 (Supplemental Data).

#### Caregiver‒child conflicts

This is a modified scale with four items. It is used to assess four areas of conflict, including decision-making, social life, academic expectations from parents, and caregivers making child-related comparisons (e.g., “Your caregiver [s] always compares you to others, but you want them to accept you for being yourself”). Items are rated on a 5-point scale from 1 (never) to 5 (always). The alpha coefficient was 0.641 (Supplemental Data).

The identity of the participants was assessed through four questions, “Who do you live with now/at 5/at 10?”, and students chose from the following five answers: A. parents; B. only fathers; C. only mothers; D. grandparents; and E. others. Household registration type was coded as “0 (rural)” or “1 (urban)”. In terms of family economic conditions, three questions based on the Family Affluence Scale^82^ were used to estimate the students’ family economic status (e.g., “Does your family own a car?”). The answer options were “No,” “Yes, one,” and “Yes, two or more.” Additionally, the number of siblings as well as rank among the siblings were included in the questionnaire: “How many children in your family?” answered by “one,” “two,” “three,” and “more than three,” and the rank was assessed with an open response question. Moreover, information about each parent or their caregivers’ age, education, and time of migration was assessed through single questions, for example, “What is your parents’ education?”, with options ranging from A. Primary School or below to D. College/University or above.

### Ethics statement

The process of my research was approved by the Examine Board of Political Science and Sociology, Philosophy Faculty, University of Bonn, Germany. According to the Circular on the Issuance of Measures for Ethical Review of Life Science and Medical Research Involving Human Beings, published on the official website of the Central People's Government of the People's Republic of China, life science and medical research involving human beings should respect and safeguard the rights of the research participants or their guardians to be informed and to make autonomous decisions regarding research participation. The procedure for informed consent was strictly followed. Additionally, the privacy and personal information of participants must be protected. This includes effectively safeguarding the research participant’s privacy, truthfully informing them about the collection, storage, use, and confidentiality of their personal information, obtaining their permission, and ensuring that their personal information is not disclosed to third parties without their authorization. The researchers of this paper strictly adhered to all the aforementioned ethics and regulations. The study included participants from China. Therefore, we obtained the Ethics Certificate from Shenzhen Technology University. The reference number for the local ethics approval is SZTUYXLL2024034.

### Consent to participate

Protecting participants' rights and interests was a priority in this study. First, the participants were fully informed, and key information about the research was provided so that they could consider it before deciding whether to participate. Second, written informed consent was obtained from the participants, parents, or guardians of those involved in this study. Informed consent clarified that participation was voluntary and that participants could withdraw at any time. Third, all the data were anonymized and kept confidential.

### Method statement

There was no experiment included in this study. The data were collected via questionnaires and interviews. All children included in this study were informed of its purpose, and participation was voluntary. For children under 16 years old, this information was relayed to their parents or legal caregiver(s). At the beginning of the survey, all the participants were informed that "the researcher will use anonymized responses for research and publicity purposes. If you agree with your anonymous responses to be used, please answer the questions. The parents or legal guardians of children under 16 were informed. All methods were carried out in accordance with the relevant guidelines and regulations of the Declaration of Helsinki.

## Family arrangement and social cognitive wellbeing (SCWB)

The following tables show the means and standard deviations of Likert scale items in the questionnaire from participants from both inbound (most are urban areas) and outbound (most are rural areas) areas. To simplify the model testing complexity and compare the data across the three groups (students living with both parents, living with one parent, and living with others or alone), some variables were separated into sub-variables.

There are eight different variables included in the SCWB: Lifelong Satisfaction, Academic Satisfaction, Self-Efficacy, Outcome Expectations, Goal Progress, Positive & Negative Affect and Environmental Support. Each item had three to six Likert scale items for the students to answer the questionnaire, as shown in the Appendix I. One thing to be noticed is that the Self- Notably, the self-construal variables, divided into independence and interdependence, were eliminated, given that their Cronbach’s alpha values indicated that these variables were not significant for this research. Therefore, this research deleted them.

Table 5 shows the five items in the lifelong satisfaction scale, the factor loadings obtained through principal component analysis (PCA) and Cronbach’s alpha coefficients. There are five questions for this scale, with responses ranging from 1 (strongly disagree) to 5 (strongly agree).

**Table 5:** Analysis of Lifelong Satisfaction I.

Lifelong satisfaction items	Factor loading
LS1. In most ways my life is close to my ideal	0.814
LS2. The conditions of my life are excellent	0.812
LS3. I am satisfied with my life	0.813
LS4. Thus far, I have gotten the important things I want in life	0.729
LS5. If I could live my life over, I would change almost nothing	0.598
Cronbach’s Alpha: 0.805	Explained Variance: 57.4%

The KMO (Kaiser–Meyer–Olkin) value obtained for the five items was 0.799, indicating that the variable is suitable for principal component analysis. According to the eigenvalue criterion (eigenvalue = 1), one common factor was extracted from the five items that explained 57.4% of the total variance. The variables that correlated the most with the principal component (D1) were LS1: *“In most ways, my life is close to my ideal*” (0.814); LS2: *“The conditions of my life are excellent”* (0.812); and LS3: *“I am satisfied with my life”* (0.813). The principal component is positively correlated with all five of these items. Therefore, increasing the values of LS1, LS2, LS3, LS4 and LS5 improved the value of the principal component (*Lifelong Satisfaction*). It increases the most when a participant strongly agrees with the item *“In most ways, my life is close to my ideal*” (LS1). The Cronbach’s alpha was 0.805, indicating the results were sufficiently reliable.

Table 6 shows the statistical analyses of these lifelong satisfaction variables, including the means, standard deviations, eta (η), standard deviation ($\sigma$), and statistical significance (p-value), through ANOVA.

**Table 6:** Analysis of Lifelong Satisfaction II.

Living with		LS1	LS2	LS3	LS4	LS5
Both parents	$\overline{\text{x} }$:	2.88	3.19	3.17	2.43	2.32
n: 1,054–1,063	$\upsigma$:	0.941	0.960	0.973	0.986	1.133
One parent	$\overline{\text{x} }$:	2.83	3.08	3.09	2.42	2.23
n: 304–307	$\upsigma$:	0.913	0.985	1.035	0.995	1.087
Others/alone	$\overline{\text{x} }$:	2.78	3.08	3.01	2.40	2.08
n: 305–306	$\upsigma$:	0.974	0.994	1.015	1.045	1.136
Total	$\overline{\text{x} }$:	2.85	3.15	3.12	2.42	2.26
n: 1,664–1,676	$\upsigma$:	0.942	0.972	0.993	0.998	1.128
$\upeta$:		n.s	n.s	0.063	n.s	0.078
P Value:		n.s	n.s	< 0.05	n.s	< 0.05

An ANOVA test was used to compare the means of Lifelong satisfaction items from three groups that identified family arrangements and who participants lived with. There was a statistically significant difference only for LS3 and LS5 (P < 0.05). The effect size of LS5 is the largest between the two variables ($\eta =$ 0.078), followed by that of LS3 ($\eta =0.$ 063).

For LS3, “*I am satisfied with my life”*, the mean score was 3.17 for participants living with both parents, 3.09 for participants living with one parent and 3.01 for participants living with others or alone; all the mean scores were slightly above 3 (neither agree nor disagree). For LS3, the standard deviation was 0.973 for participants living with both parents and 1.035 and 1.015 for those living with one parent and other conditions, respectively. The fluctuation for participants living with both parents is smaller than that of other conditions. For the fifth question, LS5 “*If I could live my life again, I would change almost nothing”,* the mean for participants living with both parents was 2.32, with one parent, 2.23, and with others or alone, 2.08. All groups were nearly 2 (disagree), and the standard deviations were 1.133 for participants living with both parents, 1.087 for participants living with one parent and 1.128 for participants living with others or alone. In summary, participants who lived with both parents had slightly greater average scores than those in the other two groups.

Table 7 shows that the *Academic Satisfaction* items are also measured on Likert scales ranging from 1 (strongly disagree) to 5 (strongly agree). These data reflect the participants’ satisfaction with their academic life and school. Additionally, PCA was used to determine the factor loading.

**Table 7:** Analysis of Academic Satisfaction I.

Academic satisfaction	Factor loading
AS1. I am comfortable with the educational atmosphere in my major field	0.802
AS2. For the most part, I am enjoying my coursework	0.800
AS3. I am generally satisfied with my school life	0.761
AS4. I enjoy the level of intellectual stimulation in my courses	0.778
AS5. I feel enthusiastic about the subject matter in my intended major	0.834
AS6. I like how much I have been learning in my classes	0.798
Cronbach’s Alpha: 0.884	Explained variance: 57.4%

The KMO value for the six items was 0.767, implying that the factor is suitable for PCA. According to the eigenvalue criteria, one common factor was extracted from the six factors that explained 57.4% of the total variance. Items AS1: *“I am comfortable with the educational atmosphere in my major field”*, AS2: *“For the most part, I am enjoying my classes”* and AS5: *“I feel enthusiastic about the subject matters in my major”* correlate the most with the principal component (D1), with factor loadings of 0.802, 0.800 and 0.834, respectively. In addition, the principal component is positively correlated with all six of these items. Therefore, increasing the value of these six items improved the value of the principal component (academic satisfaction). In particular, for AS5, when the participants study in the major they are interested in, it is most helpful to improve their academic satisfaction. Cronbach’s alpha was 0.884, indicating that the symbols of the common factor have good reliability.

Table 8 shows information on the Academic Satisfaction items. All six items had P < 0.05, indicating statistical significance for all variables. The means of all five questions were between 3 (Neither agree nor disagree) and 4 (Agree) among the three groups. For the Eta values, the effect size of the AS5 was the largest among the six Items $\upeta =0.10$ 3, followed by the effect size of the AS2, $\upeta =0.$ 095. The remaining Eta values are similar (0.072 $<\upeta <$ 0.103). The standard deviations of these scales range from 0.886 to 1.008. Additionally, for these academic satisfaction items, the averages for participants living with both parents are generally greater than those for the other two groups. This indicates that participants living with both parents possess higher levels of academic satisfaction.

**Table 8:** Analysis of Academic Satisfaction II.

Living with		AS1	AS2	AS3	AS4	AS5	AS6
Both parents	$\overline{\text{x} }$:	3.43	3.21	3.34	3.54	3.36	3.50
n: 1055–1061	$\upsigma$:	0.886	0.931	0.942	0.908	0.911	0.903
One parent	$\overline{\text{x} }$:	3.35	3.06	3.18	3.37	3.18	3.47
n: 302–307	$\upsigma$:	0.889	0.974	1.008	0.951	1.002	0.974
Others/alone	$\overline{\text{x} }$:	3.26	3.00	3.20	3.37	3.14	3.32
n: 304–306	$\upsigma$:	0.967	0.970	0.984	1.004	0.983	0.918
Total	$\overline{\text{x} }$:	3.39	3.15	3.28	3.48	3.29	3.46
n: 1661–1674	$\upsigma$:	0.904	0.950	0.964	0.938	0.947	0.921
$\upeta$:		0.074	0.095	0.074	0.089	0.103	0.072
P Value:		< 0.05	< 0.01	< 0.05	< 0.01	< 0.001	< 0.05

The third SCWB factor tests the self-efficacy of the participants. The items were given a 5-item scale (shown in Table 9) ranging from 1 (no confidence) to 5 (complete confidence).

**Table 9:** Analysis of Self-Efficacy I.

Self-efficacy	Factor loading
SE1. Remain enrolled in your intended major over the next semester	0.684
SE2. Excel in your intended major over the next semester	0.397
SE3. Complete the upper level required courses in your intended major with an overall grade point average of 70 points or better (100 is the best score)	0.782
SE4. Find ways to avoid communication problems with teachers and teaching assistants in your courses	0.798
SE5. Balance the pressures of studying with the desire to have leisure time for fun and other activities	0.773
Cronbach’s Alpha: 0.727	Explained variance: 74.7%

Based on the PCA results, the KMO value for the five items is 0.767, indicating that the variable is suitable for PCA. According to the eigenvalue criteria, one common factor was extracted from the four factors that explained 74.66% of the variance in the items. All items positively reflect the principal component, and the items SE3: *“To complete the upper-level required courses in my intended major with an overall grade point average of 70 or higher (100 being the best score)”* (0.782), SE4: *“To find ways to avoid communication problems with teachers and teaching assistants in my courses”* (0.798) and SE5: *“To balance the pressures of studying with the desires to have time for fun and other activities”* (0.773) correlate the most with the principal component (D1). This means that avoiding communication problems with teachers contributes most to the principal component of self-efficacy. Furthermore, Cronbach’s alpha was 0.727, indicating that the symbols of the common factor have sufficient reliability.

Table 10 shows the ANOVA test results for the *Self-Efficacy* scale. Three variables had P values less than 0.05, indicating substantial significance. For the Eta values, the effect size of SE3 is the largest among the five variables (0.107), and that of SE2 is the smallest, at $\upeta =0.$ 064. This factor showed similar results to those for *Lifelong satisfaction* and *Academic Satisfaction*, and the mean number of answers from participants living with both parents was generally greater than that of participants in the other two groups. Since *Self-Efficacy* refers to the personal belief of how competent one could be when solving or completing a particular task, this shows that students living with both parents have greater confidence in themselves than those from the other two groups. The highest mean was 3.02, and the lowest was 1.90.

**Table 10:** Analysis of Self-Efficacy II.

Living with		SE1	SE2	SE3	SE4	SE5
Both parents	$\overline{\text{x} }$:	3.01	2.07	2.69	2.60	3.02
n: 1061–1064	$\upsigma$:	1.039	1.059	1.058	1.030	1.037
One parent	$\overline{\text{x} }$:	2.95	1.98	2.54	2.44	2.89
n: 302–307	$\upsigma$:	1.047	1.064	1.113	1.002	1.142
Others/alone	$\overline{\text{x} }$:	2.97	1.90	2.41	2.45	2.89
n: 304–306	$\upsigma$:	1.101	1.025	0.942	1.014	1.081
Total	$\overline{\text{x} }$:	2.99	2.03	2.61	2.55	2.97
n: 1667–1676	$\upsigma$:	1.052	1.055	1.054	1.024	1.066
$\upeta$:		n.s	0.064	0.107	0.075	n.s
P Value:		n.s	< 0.05	< 0.01	< 0.05	n.s

Table 11 shows the PCA results and the Cronbach’s alpha coefficient of the *Outcome Expectations* scale, a 5-item scale with answers ranging from 1 (strongly disagree) to 5 (strongly agree). The KMO value for the five items was 0.854, implying that the variable is suitable for PCA. According to the eigenvalue criteria, one common factor is extracted from the five that explains 71.9% of the item variance. The variables most strongly correlated with the principal component (D1) are OE2: *“earn an attractive salary”* (0.889), OE1: *“receive a good job offer (or graduate school) offer”* (0.867), OE3: *“be respected by other people”* (0.867), OE4: *“increase my sense of self-worth”* (0.839) and OE5: *“have a career that is valued by my family”* (0.775). This indicates that earning a good salary after graduation contributes most to the principal component, followed by receiving a good job and respect from other people. In addition, the principal component is positively correlated with all five variables. The Cronbach’s alpha was 0.902, indicating that the symbols of the common factor have excellent reliability.

**Table 11:** Analysis of Outcome Expectations I.

Outcome expectations	Factor loading
Graduating in vocational school will likely allow me to:	
OE1. receive a good job (or graduate school) offer	0.867
OE2. earn an attractive salary	0.889
OE3. get respect from other people	0.867
OE4. increase my sense of self-worth	0.839
OE5. have a career that is valued by my family	0.775
Cronbach’s Alpha: 0.902	Explained variance: 71.9%

ANOVA test results for the outcome expectation scale*.* The mean, standard deviation, Eta, reliability coefficients and factor loading of these SCWB factors are shown in Table 11. All items had P > 0.05, indicating no statistical significance for the outcome expectation variable in determining the group of participants.

Table 12 shows the *Goal Progress* scale. The base question for this Likert scale was *“How much progress are you making towards each of these goals at this point,* i.e.*, thus far this semester?”,* followed by four items of students’ goal progress, with responses ranging from 1 (no progress) to 5 (excellent progress). The result of the KMO test was 0.817 for the factor, and the chi-square value of the Bartlett sphere test was statistically significant, showing that the items are fit for CPA. Based on the given value criteria, one common factor is extracted from the four that explains 74.66% of the variance. The variables that correlate the most with the principal component (D1) are GP2, *“I have good marks in all of my exams”* (0.869), and GP3, *“I am achieving/maintaining high marks/grades in all of my courses”* (0.898). The principal component is positively correlated with all four of these items. Additionally, Cronbach’s alpha is 0.886, indicating the factor has excellent reliability.

**Table 12:** Analysis of Goal Progress I.

Goal progress	Factor loading
How much progress are you making towards each of these goals at this point in time (i.e., thus far this semester):	
GP1. Completing all course assignments in time	0.839
GP2. Have good marks in all of my exams	0.869
GP3. Achieving/maintaining high marks/grades in all of my courses	0.898
GP4. Learning and understanding the contents in each of my courses	0.849
Cronbach’s Alpha: 0.886	Explained variance: 74.7%

Table 13 shows the information for the *Goal Progress* SCWB factor. There are significant differences for all the *Goal Progress* items, which all have P < 0.001, which indicates substantial differences for participants who live together with parents for all four items. The participants who lived with both parents had higher averages for this factor. The eta values show that the effect size of each item is greatest for GP2, “*I have good marks on all of my exams”* ($\upeta$= 0.175). The means of the items in the three groups varied between 2.57 and 3.26. The average number of participants living with both parents is relatively greater for each item than for the other two groups since goal progress refers to how much progress the students have made in achieving their goal of studying in VET schools. Students living with both parents have better perceptions of the progress they have made in achieving their academic goals than those who live with others.

**Table 13:** Analysis of Goal Progress II.

Living with		GP1	GP2	GP3	GP4
Both parents	$\overline{\text{x} }$:	3.11	3.26	2.91	3.11
n: 1064–1065	$\upsigma$:	0.965	1.046	1.003	0.987
One parent	$\overline{\text{x} }$:	2.82	2.90	2.65	2.88
n: 307	$\upsigma$:	1.039	1.140	1.069	1.018
Others/Alone	$\overline{\text{x} }$:	2.80	2.83	2.57	2.70
n: 306	$\upsigma$:	1.013	1.088	1.029	1.002
Total	$\overline{\text{x} }$:	3.00	3.12	2.80	3.00
n: 1,677–1,678	$\upsigma$:	1.001	1.091	1.043	1.023
$\upeta$:		0.143	0.175	0.143	0.161
P value:		< 0.001	< 0.001	< 0.001	< 0.001

Table 14 shows the content of the *Positive Affect* Scale, which reveals the participants’ positive emotional disposition, with answers ranging from 1 (never) to 5 (always). In addition, it also includes the results of PCA and Cronbach’s alpha coefficient. The result of the KMO test for these items was 0.768, and the chi-square value of the Bartlett sphere test was statistically significant, which shows that the items are fit for a PCA. The principal component accounts for 49.7% of the total variance and is positively correlated with all five items. Therefore, increasing the values of these items slightly increases the value of the principal component of *Positive Affect*. In addition, the items that correlated the most with the principal component were NPA5: *“Enthusiastic”* (0.769) and NPA11*: “Active”* (0.722), while NPA6: *“Proud”* (0.648) had the lowest correlation. In addition, the Cronbach’s alpha is 0.747, showing that the scale has sufficient reliability.

**Table 14:** Analysis of Positive Affect I.

Positive affect	Factor loading
How often do you feel:	
NPA2: Excited	0.690
NPA5: Enthusiastic	0.769
NPA6: Proud	0.648
NPA9: Inspired	0.691
NPA11: Active	0.722
Cronbach’s Alpha: 0.747	Explained variance: 49.7%

The ANOVA test shows the positive affect scale. There was no significant difference for any of the five items of the positive affect subitems; they all had P values greater than 0.05, indicating no significant difference between participants from different groups in terms of their *Positive Affect*.

Table 15 shows the items on the *Negative Affect* Scale, which reveals the negative emotional disposition of the participants, with answers ranging from 1 (never) to 5 (always). Additionally, it includes the results of PCA and Cronbach’s alpha coefficient. The result of the KMO test of the *Negative Affect* subitems was 0. 887, and the chi-square value of the Bartlett sphere test is statistically significant, which shows that the variables are fit for PCA. The principal component accounted for 53.2% of the total variance. The items that correlated the most with the principal component were NA3: “*Anxiety*” (0.784) and NA7: *“Irritable”* (0.759). This indicates that when participants feel more anxious and irritable, this helps improve the principal component of negative affect. Additionally, Cronbach's coefficient was 0.852, showing excellent reliability for this SCWB factor.

**Table 15:** Analysis of Negative Affect I.

Goal progress	Factor loading
How often do you feel:	
NPA1: Distressed	0.750
NPA3: Anxiety	0.784
NPA4: Lonely	0.727
NPA7: Irritable	0.759
NPA8: Ashamed	0.676
NPA10: Nervous	0.654
NPA12: Afraid	0.747
Cronbach’s Alpha: 0.852	Explained variance: 53.2%

Table 16 shows the *Negative Affect* subitems; most had P values smaller than 0.05 (NPA4, NPA7, NPA10 and NPA12), indicating significant differences for participants across these three groups. In contrast, there was no significant difference among the participants in the different groups for NPA1, NPA3 or NPA8. NPA4 (“*Lonely”*), NPA7 (*“Irritable”*), NPA10 (*“Nervous”*) and NPA12 (*“Afraid”*) are negative emotions, and this study revealed that participants living with others or alone generally have more *Negative Affect* and emotions.

**Table 16:** Statistical Analysis of Negative Affect.

Living with		NPA1	NPA3	NPA4	NPA7	NPA8	NPA10	NPA12
Both parents	$\overline{\text{x} }$:	2.46	2.78	2.70	2.92	2.51	3.02	2.59
n: 1,061–1,064	$\upsigma$:	0.884	0.954	1.047	0.950	0.874	0.913	0.985
One parent	$\overline{\text{x} }$:	2.47	2.84	2.86	3.04	2.56	2.97	2.68
n: 304–307	$\upsigma$:	0.933	0.996	1.128	0.979	0.860	0.933	1.094
Others/alone	$\overline{\text{x} }$:	2.58	2.92	3.03	3.08	2.60	3.14	2.83
n: 302–06	$\upsigma$:	0.935	0.939	1.138	0.948	0.872	0.928	1.094
Total	$\overline{\text{x} }$:	2.48	2.81	2.79	2.97	2.54	3.03	2.65
n: 1,670–1,676	$\upsigma$:	0.903	0.960	1.086	0.958	0.871	0.921	1.030
$\upeta$:		0.050	0.058	0.120	0.070	0.040	0.060	0.090
P Value:		n.s	n.s	< 0.01	< 0.05	n.s	< 0.05	< 0.01

Positive affect is not significant, but negative affect is among the three groups (live with both parents, live with one parent and live with others or alone), which may imply that positive emotions come from inner expectations, while negative emotions come from external influences.

Table 17 shows the PCA results and Cronbach’s alpha coefficient of the *Environmental Support* Scale, a five-item scale with answers ranging from 1 (strongly disagree) to 5 (strongly agree). The result of the KMO test was 0.688, the chi-square value of the Bartlett sphere test was considerable, and it was statistically significant; this shows that the variables are fit for a CPA. According to the principle of eigenvalue, which has results greater than 1, one common factor is extracted from the three items that explain 66.5% of the total variance. The variables that correlated the most with the principal component were ESP1 (0.834) and ESP3 (0.812). The principal component is positively correlated with all three of these variables. In particular, support from friends contributes most to the principal component. Additionally, Cronbach’s alpha was 0.747, indicating the scale has satisfactory reliability. The factor loading coefficients suggest that the *environmental support* items have similar weights in this SCWB factor.

**Table 17:** Analysis of Environmental Support I.

Environmental support	Factor loading
At the present time, I …	
ESP1. Get encouragement from my teachers for pursuing my intended major	0.812
ESP2. Feel that my family members support the decision to major in my intended field	0.800
ESP3. Feel that close friends would be proud of me for majoring in my intended field	0.834
Cronbach’s Alpha: 0.747	Explained variance: 66.5%

Table 18 shows the ANOVA test results for the *Environmental Support* Scale. The means, standard deviations, and ETA for the *Environmental Support* items are shown in the table above. Since the participants were students, this included support from parents or other caregivers, teachers and classmates. Two of the three items (ESP1: *“I get encouraged by my teachers to pursue my major”*; ESP3: *“I feel that my close friends support my decision to major in my field”*) have significant differences across groups, but ESP2 does not (*I feel that my family members would be proud of me for majoring in my intended field”*). This shows that encouragement from teachers and close friends is significantly different among participants with different caregivers. There are generally greater means for participants living with both parents, which shows that participants living with both parents feel more support from teachers and close friends than other groups.

**Table 18:** Analysis of Environmental Support II.

Living with		ESP1	ESP2	ESP3
Both parents	$\overline{\text{x} }$:	3.50	3.69	3.61
n: 1,062	$\upsigma$:	0.878	0.889	0.840
One parent	$\overline{\text{x} }$:	3.42	3.57	3.55
n: 306–307	$\upsigma$:	0.933	1.010	0.901
Others/alone	$\overline{\text{x} }$:	3.34	3.64	3.46
n: 306	$\upsigma$:	0.986	0.942	0.891
Total	$\overline{\text{x} }$:	3.46	3.66	3.57
n: 1,674–1,675	$\upsigma$:	0.910	0.923	0.862
$\upeta$:		0.071	0.053	0.067
P Value:		< 0.05	n.s	< 0.05

PCA was used to clarify the principal component of each SCWB variable, and ANOVA was used to compare the averages of the SCWB variables across the three student groups (living with both parents, living with one parent and living with others or alone). The P values of each test show that most SCWB variables are statistically significant for determining the caregiver(s) of the student, of which only *Outcome Expectations* and *Positive Affect* are not. Moreover, the average SCWB factor scores for participants living with both parents were generally greater than those of the other groups, and these values were inverted for the *Negative Affect* factor. For most SCWB variables, a higher mean indicates more satisfying or positive experiences or feelings (e.g., lifelong satisfaction, Academic Satisfaction, Self-Efficacy, Outcome Expectations, Goal Progress, Positive Affect and Environmental Support). For the rest of the variables in the above tables, a higher score indicates more negative expectations or experiences. The Cronbach’s alpha values of all the variables are above 0.6, indicating high reliability for all these variables.

## Empirical analysis and results

The social cognitive model of well-being^4^ is one of the few theoretical frameworks for illustrating human strengths and positive adjustment. This framework underlines the mediating functions of cognitive factors (self-efficacy, outcome expectation, and goal progress) that link affective traits/personality (positive or negative affect) and environmental factors (environmental support and obstacles) to well-being outcomes^5^. In this study, six family relationship factors (caregiver-child coactivity, caregiver-child communication frequency, caregiver-child regulation, caregiver-child conflicts, caregiver-child alienation, and caregiver-child trust communication), divided into two parts, were involved in the environmental factors of the SCWB. Structural equation modelling was used in this part to test the paths of these two new models.

### Preliminary analyses (Supplemental Data)

There was a small percentage of missing values (1.973%) across 131 variables and 1,682 participants. Despite the results for Little’s missing completely at random (MCAR) test being significant (sig < 0.05), a visual inspection of the dataset implied a generally random pattern of missing data. Missing values were inputted using an expectation–maximization algorithm in SPSS 22 (these data were used for the following two models). The hypothesis of the revised model and its paths are shown in Fig. 2, which is based on previous studies and the characteristics of Chinese migrant workers’ children.

Structural equation modelling in AMOS 22 was used to test the structural validity of these models within the target population. The bootstrap method was used to test indirect effects related to environmental support, self-efficacy, and caregiver-child coactivity; 2,000 bootstrap samples were generated to estimate the indirect effects and bias-corrected 90% confidence intervals. Here, three fit indices were used to assess the data fit: the comparative fit index (CFI), the root mean squared error of approximation (RMSEA), and the root mean squared residual (SRMR). RMSEA $\le$ 0.08, CFI $\ge$ 0.90, and SRMR $\le$ 0.08 are considered adequate fits, and RMSEA $\le$ 0.06, CFI $\ge$ 0.95, and SRMR $\le$ 0.05 are considered good fits^83^. Additionally, researchers have determined that models meeting only one of the three values (CFI, RMSEA, and SRMR) can be considered an acceptable fit^38^. The results of confirmatory factor analysis (CFA) of the above model with the current dataset show an acceptable fit, with ${x}^{2}$= 8829, df = 1621, p < 0.0001, CFI = 0.846, RMSEA = 0.051 and SRMR = 0.076.

**Figure 5:**
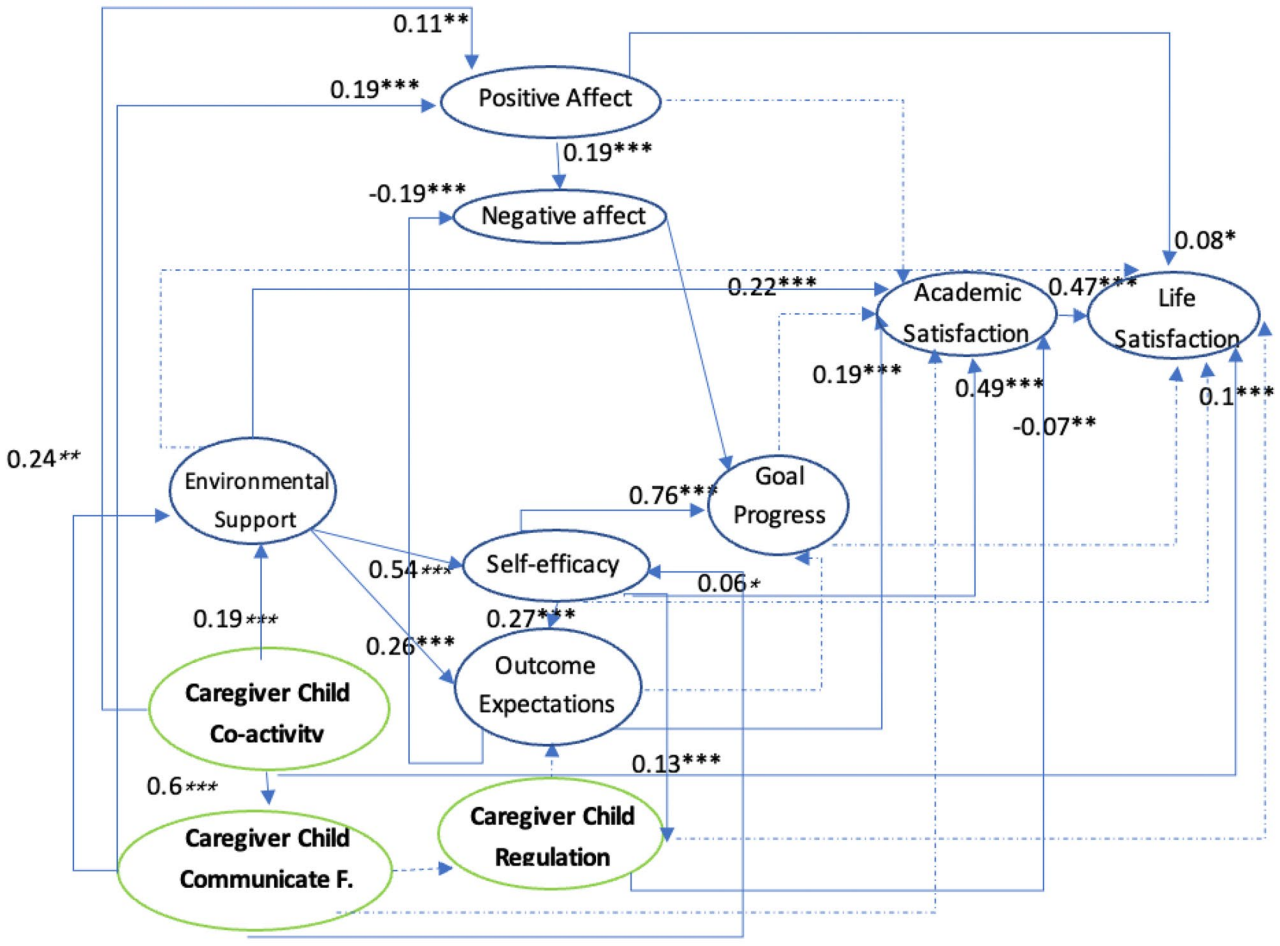
Family Relationship and Social Cognitive Model I – Path Coefficients. Communicate. Figure 5 shows the short form for caregiver-child communication frequency.

Figure 5 shows the path coefficients of family relationships and social cognitive Model I. According to the results of structural equation modelling, positive affect and caregiver-child coactivity have indirect effects on lifelong satisfaction, and the indirect (mediated) effect of caregiver-child coactivity on positive affect is 0.114 and statistically significant (P < 0.001). Moreover, environmental support and self-efficacy have significant indirect effects on outcome expectations; self-efficacy and outcome expectations have significant indirect effects on academic satisfaction. The significant standard indirect effects for lifelong satisfaction ranged from − 0.287 to 0.283, whereas those for academic satisfaction ranged from 0.100 to 0.310.

**Figure 6:**
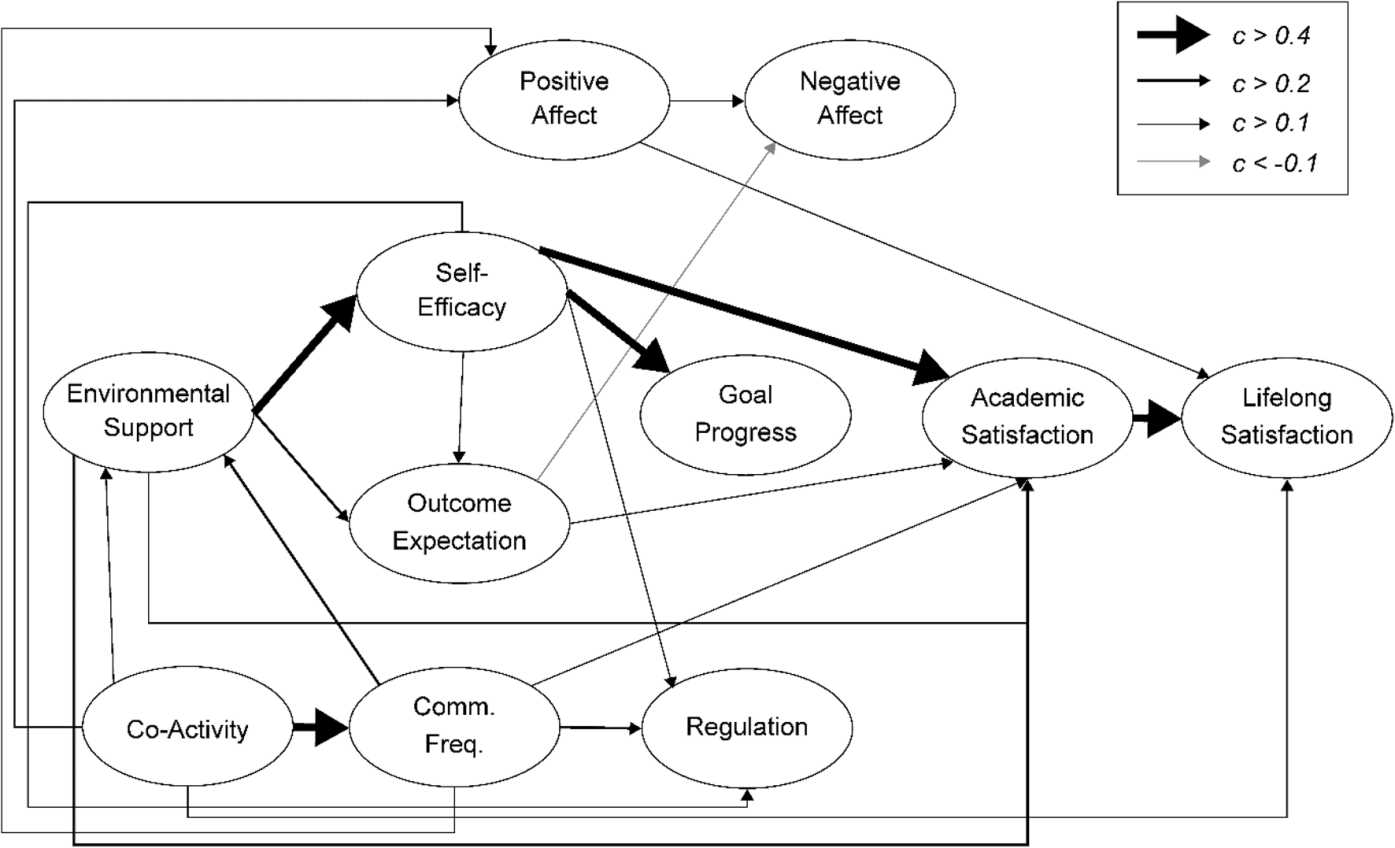
Family-Related Modified Social Cognitive Model I-Path Coefficients. Comm. Freq. Figure 6 shows the short form for caregiver-child communication frequency.

Figure 6 shows the research hypothesis that most paths are statistically significant. Moreover, most paths between SCWB variables are strongly correlated; for example, the coefficients of the paths from environmental support to self-efficacy, self-efficacy to goal progress, goal progress to academic satisfaction and academic satisfaction to lifelong satisfaction are both above 0.4. Furthermore, the family relationship variables are closely correlated; the coefficients of the paths from caregiver-child coactivity to communication frequency are above 0.4, those from communication frequency to regulation are above 0.2, and those from caregiver-child coactivity to regulation/house rules are above 0.1. Most paths are statistically significant, as predicted in Fig. 2. This finding is consistent with the results of other studies^2,38^. Moreover, the path from communication frequency to environmental support was greater than 0.2, which is easy to understand because students with better communication with caregiver(s) or parents receive more support from their caregiver(s)/parents. Unexpectedly, the path from goal progress to academic satisfaction is not significant.

The results in Table 19 show the coefficients, covariance, and p values of all paths in the research hypothesis, which are statistically significant, except for nine paths ($P$>$0.05$). The path coefficients for many social cognitive variables (from environmental support to self-efficacy, from self-efficacy to goal progress, from environmental support to outcome expectation, from goal progress to academic satisfaction, and academic satisfaction to lifelong satisfaction) showed similar results to those of previous studies^38^ related to the SCWB model. Most of the path coefficients with statistical significance were above zero, except for the path coefficient of the path from outcome expectation to negative affect ($path coeficient=-0.19,P<0.001$). The path coefficient of the path from self-efficacy to goal progress was the largest ($path coeficient=0.76,P<0.001$), which means that when self-efficacy improved by 1 unit, goal progress increased by 0.76 units, *ceteris paribus*.

**Table 19:** Significant Differences in Standardized Structure Path Coefficients Model I (Supplemental Data).

Path		Coef	P
Caregiver-child Coactivity→ Caregiver-child Comm. Fre		0.60	***
Caregiver-child Coactivity→ Environmental support		0.19	***
Caregiver-child Comm. Fre→ Environmental support		0.24	***
Caregiver-child Comm. Fre→ Self-Efficacy		0.06	*
Environmental support→ Self-Efficacy		0.54	***
Caregiver-child Comm. Fre→ Caregiver-child Regulation		0.29	***
Caregiver-child Coactivity→ Caregiver-child Regulation		0.05	*N.S*
Self-Efficacy→ Caregiver-child Regulation		0.13	***
Self-Efficacy→ Outcome Expectation		0.27	***
Caregiver-child regulation→ Outcome Expectation		0.04	*N.S*
Environmental support→ Outcome Expectation		0.26	***
Self-Efficacy→ Goal Progress		0.76	***
Caregiver-child Comm. Fre→ Positive Affect		0.19	***
Caregiver-child Coactivity→ Positive Affect		0.11	**
Outcome Expectation→ Goal Progress		0.01	*N.S*
Self-Efficacy→ Academic Satisfaction		0.49	***
Caregiver-child Regulation→ Academic Satisfaction		− 0.07	**
Caregiver-child Comm. Fre→ Academic Satisfaction		0.05	*N.S*
Outcome Expectation→ Academic Satisfaction		0.19	***
Goal Progress→ Academic Satisfaction		0.03	*N.S*
Positive Affect→ Academic Satisfaction		− 0.03	*N.S*
Environmental support→ Academic Satisfaction		0.22	***
Academic Satisfaction→ Lifelong satisfaction		0.47	***
Outcome Expectation→ Negative Affect		− 0.19	***
Self-Efficacy→ Lifelong Satisfaction		0.02	*N.S*
Positive Affect→ Lifelong Satisfaction		0.08	*
Goal Progress→ Lifelong Satisfaction		0.00	*N.S*
Environmental Support→ Lifelong Satisfaction		0.07	*N.S*
Positive Affect→ Negative Affect		0.19	***
Caregiver-child Regulation→ Lifelong satisfaction		0.02	*N.S*
Caregiver-child Coactivity→ Lifelong satisfaction		0.11	***

*Comm. Fre.* communication frequency, *C.R.* covariance.

* < 0.05, ** < 0.01, *** < 0.001.

This is followed by the path from caregiver-child coactivity to caregiver-child communication frequency ($$path coeficient=0.60, P<0.001$$), the path from environmental support to self-efficacy ($$path coeficient=0.54, P<0.001$$), the path from self-efficacy to academic satisfaction ($$path coeficient=0.49,P<0.001$$), and the path from academic satisfaction to lifelong satisfaction ($$path coeficient=0.47,P<0.001)$$. Moreover, the path from caregiver-child coactivity to caregiver-child regulation, the path from caregiver-child regulation to outcome expectations, and another nine paths were not significant. Furthermore, many paths with statistical significance are between caregiver-child relationship variables and SCWB variables, for example, the path from caregiver-child coactivity to positive affect ($$path coeficient=011,P<0.001$$), the path from self-efficacy to caregiver-child regulation ($$path coeficient=0.13,P<0.001$$), the path from caregiver-child communication frequency to positive affect ($$path coeficient=0.19,P<0.001$$), and the path from caregiver-child coactivity to lifelong satisfaction ($$path coeficient=0.11, P<0.001$$). This finding implies that more communication and coactivities between a child and caregiver(s) can improve the quality of self-efficacy, positive affect, and lifelong satisfaction of Chinese VET school students. Caregiver–child regulation was negatively correlated with academic satisfaction but positively correlated with outcome expectations.

Figure 3 shows the research hypothesis of the second family relationship SCWB model, which included two caregiver-child attachment variables (caregiver-child communication and trust and alienation) and caregiver-child conflict. The paths between social cognitive well-being variables were based on previous SCWB studies^37,38^. The paths between the SCWB variables and family relationship variables were based on the characteristics of Chinese VET school students from migrant families. According to our hypothesis model, self-efficacy, outcome expectations, goal progress, and academic satisfaction bridged the correlation between environmental support and lifelong satisfaction.

**Figure 7:**
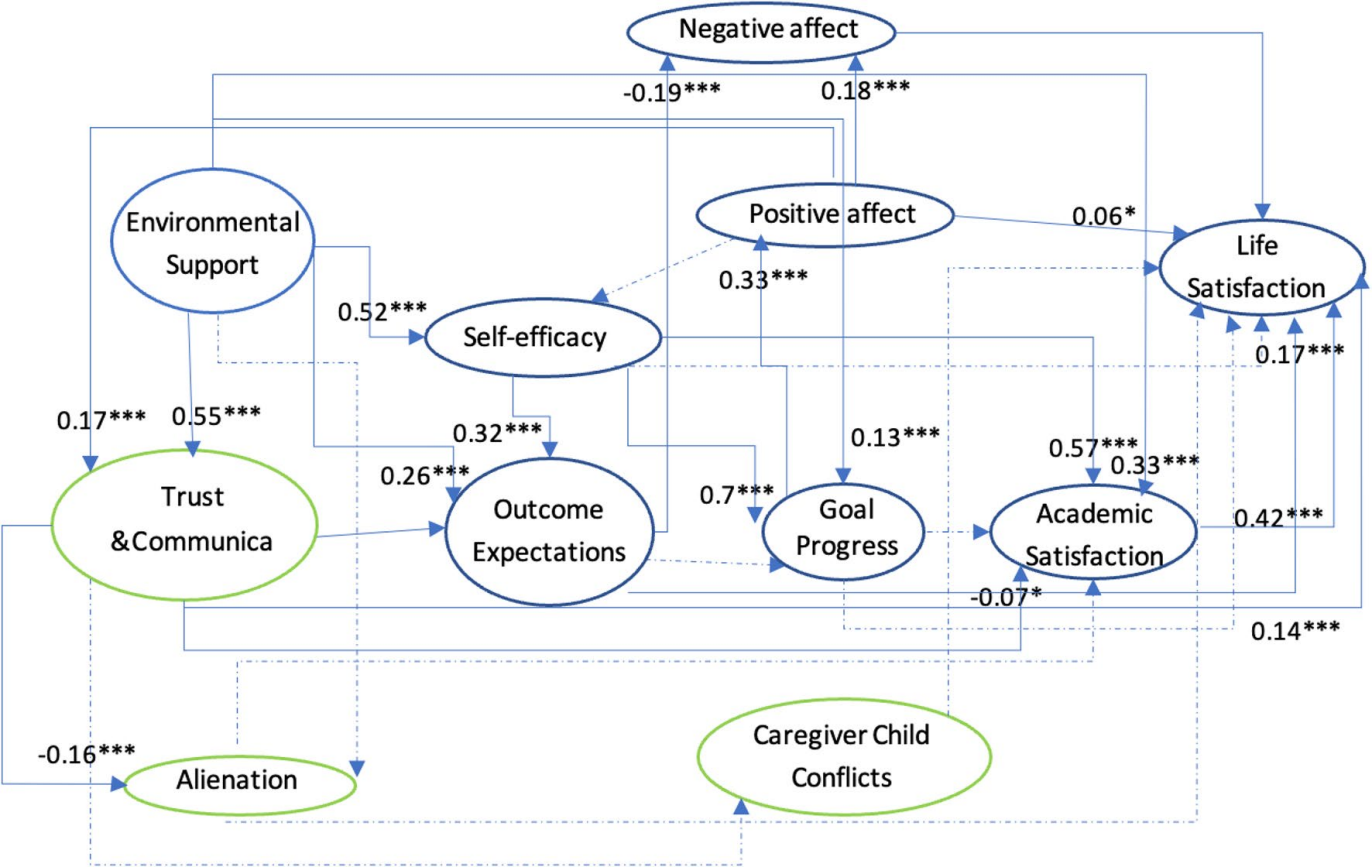
Family Relationship and Social Cognitive Model II – Path Coefficients. Trust & communica. are short for caregiver-child trust and communication, alienation is short for caregiver-child alienation, and conflict is short for caregiver-child conflict, as shown in Fig. 2.

**Figure 8:**
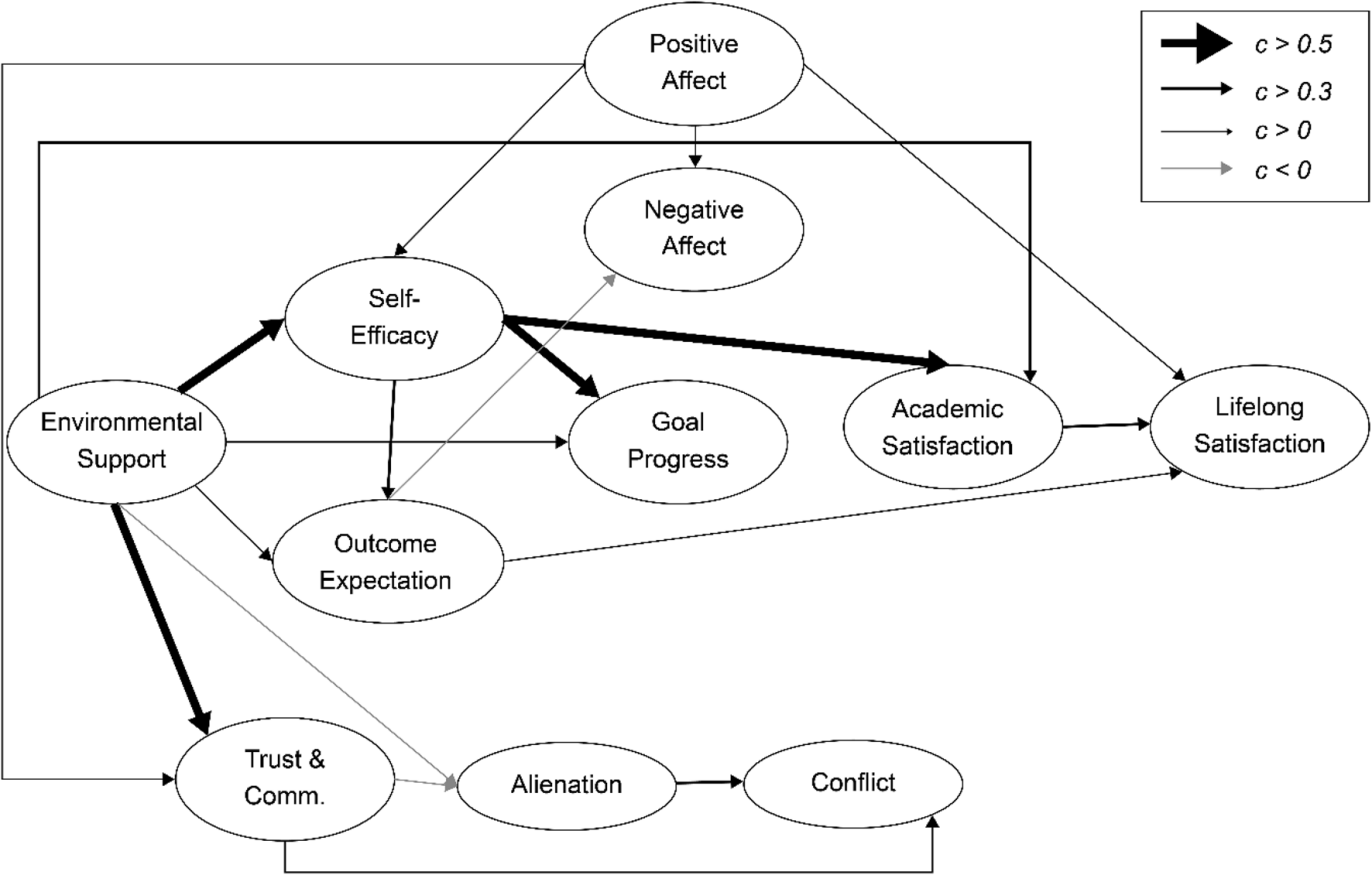
Family Relationship and Social Cognitive Model II-Path Coefficients. Trust and comm. are short for caregiver-child trust and communication, Alienation is short for caregiver-child alienation, and conflict is short for caregiver-child conflict, as shown in Fig. 8.

Figure 7 shows the path coefficients of family relationships and the social cognitive model II. The structural equation modelling analysis results for the child-caregiver attachment and conflict social cognitive model with the current data also show an acceptable fit, with ${x}^{2}$= 7576.2, df = 1740, p < 0.0001, CFI = 0.861, RMR = 0.068 and RMSEA = 0.045. The coefficients of the four significant paths above 0.5 (shown in Fig. 6) are the path from environmental support to caregiver-child trust and communication, the path from environmental support to self-efficacy, the path from self-efficacy to goal progress, and the path from self-efficacy to academic satisfaction. Moreover, the coefficients of the other four paths are above 0.3, which includes the path from environmental support to academic satisfaction, the path from self-efficacy to outcome expectations, the path from academic satisfaction to lifelong satisfaction, and the path from alienation to conflicts. Moreover, four of the significant paths are less than 0, which means that the correlation between these two variables is negative, for example, the path from environmental support to alienation, the path from outcome expectations to negative affect, the path from trust and communication to alienation and the path from alienation to lifelong satisfaction. Furthermore, the coefficients of the other paths in this model are greater than 0 (Fig. 8).

Here, the bootstrap method was used to test indirect effects related to environmental support, positive affect, caregiver-child alienation, and caregiver-child trust and communication; two thousand bootstrap samples were generated to estimate the indirect effects and bias-corrected 90% confidence intervals. Positive affect and environmental support have indirect effects on lifelong satisfaction, and the indirect (mediated) effect of environmental support on positive affect is 0.104 and is statistically significant (P < 0.01). Moreover, environmental support and caregiver-child trust and communication had indirect effects on alienation; the indirect effect of environmental support on caregiver-child alienation was − 0.184 and was statistically significant (P < 0.01). Moreover, similar to Model 1, environmental support and self-efficacy had significant indirect effects on outcome expectations, and self-efficacy and outcome expectations had significant indirect effects on academic satisfaction. The significant standard indirect effects for environmental support ranged from − 0.184 to 0.369, whereas those for caregiver-child alienation ranged from − 0.184 to 0.017.

Table 20 shows the coefficients of all the significant paths in the above model. Among all the path coefficients, nine paths were not significant, and the others were statistically significant.

**Table 20:** Significant Differences in Standardized Structure Path Coefficients Model II (Supplemental Data).

Path		Coef	P
Environmental support→ Self-efficacy		0.52	***
Environmental support→ Goal progress		0.13	***
Self-efficacy→ Academic satisfaction		0.57	***
Positive affect→ Trust & communication		0.17	***
Self-efficacy→ Outcome expectation		0.32	***
Environmental support→ Alienation		0.00	*N.S*
Environmental support→ Trust & communication		0.55	***
Environmental support→ Outcome expectation		0.26	***
Goal progress→ Academic satisfaction		− 0.01	*N.S*
Environmental support→ Academic satisfaction		0.33	***
Academic satisfaction→ Lifelong satisfaction		0.42	***
Outcome expectation→ Negative affect		− 0.19	***
Trust & communication→ Parent‒child conflicts		0.09	**
Alienation→ Parent‒child conflicts		0.40	***
Self-efficacy→ Lifelong satisfaction		− 0.01	*N.S*
Outcome expectation→ Lifelong satisfaction		0.17	***
Positive affect→ Lifelong satisfaction		0.06	*
Goal progress→ Lifelong satisfaction		0.01	*N.S*
Alienation→ Lifelong satisfaction		− 0.05	*N.S*
Positive affect→ Negative affect		0.18	***
Trust & communication→ Lifelong satisfaction		0.14	***
Parent‒child conflicts→ Lifelong satisfaction		0.01	*N.S*
Goal progress→ Positive affect		0.33	***
Self-efficacy→ Goal progress		0.70	***
Positive affect→ Self-efficacy		0.05	*N.S*
Outcome expectation→ Goal progress		− 0.04	*N.S*
Alienation→ Academic satisfaction		0.00	*N.S*
Trust & communication→ Academic satisfaction		− 0.07	*
Trust & communication→ Alienation		− 0.16	***

*C.R.* covariance.

* < 0.05, ** < 0.01, *** < 0.001.

Similar to the previous model, most path coefficients between SCWB variables were significant. Among all the paths, the path from self-efficacy to goal progress had the greatest coefficient ($path coeficient=0.704, P<0.001$), followed by the path coefficient from self-efficacy to academic satisfaction ($path coeficient=0.57, P<0.001$), the path coefficient from environmental support to caregiver-child trust and communication ($path coeficient=0.55, P<0.001$), the path coefficient from environmental support to self-efficacy ($path coeficient=0.52, P<0.001$), the path coefficient from academic satisfaction to lifelong satisfaction ($path coeficient=0.42, P<0.001$), the path coefficient from environment support to academic satisfaction ($path coeficient=0.33, P<0.001$), and the path coefficient from goal progress to positive affect ($path coeficient=0.33, P<0.001$). Moreover, several variables were negatively correlated with each other, and they had path coefficients below 0, for example, the path coefficient from outcome expectations to negative affect ($path coeficient=-0.19, P<0.001$), the path coefficient from caregiver-child trust and communication to academic satisfaction ($path coeficient=-0.07, P<0.05$), and the path coefficient from caregiver-child trust and communication to alienation ($path coeficient=-0.16, P<0.001$).

Additionally, most social cognitive variables can influence lifelong satisfaction directly (positive affect, academic satisfaction and outcome expectation) or indirectly through other variables, such as academic satisfaction/outcome expectation/goal progress/self-efficacy. For the caregiver-child relation variables added to the SCWB model, caregiver-child trust and communication can positively impact environmental support and positive affect.

The measurement model tested two structural model relations hypothesized in the previous stage. First, caregiver-child communication frequency, caregiver-child regulation, caregiver-child conflicts, caregiver-child trust and communication and coactivity were positively correlated with participants’ SCWB. Second, caregiver-child alienation negatively mediated the correlations with participants’ social cognitive factors through caregiver-child trust and communication. Moreover, this study revealed that family-related variables (caregiver-child regulation, caregiver-child coactivities, caregiver-child communication frequency, caregiver-child alienation, caregiver-child conflicts and caregiver-child trust and communication) have interrelationships with SCWB variables (academic satisfaction, outcome expectation, goal progress, lifelong satisfaction, environmental support, positive affect, negative affect and self-efficacy) that can be adjusted.

## Discussion and conclusion

The family relationship indicators were included in the social cognitive model to predict how family relationships can adjust SCWB in a collectivist social background (with half of the participants from migrant families). The dataset was collected using modified SCWB models based on the theoretical logic of previous research^4,42^ and adjusted to accommodate the Chinese societal perspective. The results and findings of this study provided initial evidence for modified family-related SCWB instruments, particularly within a collectivist context. However, further exploration of other aspects, such as school or work relationships and social relations, is warranted. Given the limited research on environmental support and social relations elements, this study is the first to explore the validity of family-related SCWB instruments. It serves as an example and provides additional evidence for measurement, including environmental-related indicators, facilitating future exploration in collectivist contexts. The outcomes of this study supported most of the research hypotheses, demonstrating a good fit for the data structure model. Additionally, this study revealed that most paths in both models are statistically significant. This study also found empirical support for most paths in the two models. Self-efficacy is significantly related to adolescents’ goal progress, academic satisfaction, and outcome expectations. This finding is consistent with prior studies^5,45,76^.

Contrary to the researcher’s expectations, caregiver-child regulation and caregiver-child conflicts were positively correlated with most SCWB indicators. This may be because teenagers regard caregiver-child regulation and relevant conflicts between them and their parents as proof that adults care about them. This study noted the importance of parent–child and caregiver-child relations for teenage students in China. It is appropriate to compare migrant children with their nonmigrant fellows (left-behind children) in rural areas^14^. Several studies have shown that children left behind by both parents are more prone to suffer from negative influences than any other kind of child^84^. Similar to other studies^27^, participants who had better communication with both parents had greater SCWB than those who had better communication with only one parent or with neither of their parents.

Another unexpected result was that goal progress in both models did not produce a significant path to academic satisfaction. Although this finding contradicts that of Lent et al.^45^ in the American context, it is consistent with the results of Sheu et al*.*^41^, who included samples from Singaporeans.

Moreover, the expectation that graduates from VET schools would significantly predict academic satisfaction in Model I and lifelong satisfaction in Model II. Additionally, support from peer teenagers and teachers played an important role in self-efficacy, outcome expectations, and academic satisfaction for adolescents from migrant families in Model I. This was also significant for caregiver-child trust and communication in Model II.

In Model I, caregiver-child coactivity was significantly related to caregiver-child communication frequency, adolescents’ lifelong satisfaction, and environmental support, which is similar to the findings of Zhao et al.^13^. This implies that more coactivity between adolescents from migrant families and parents or caregivers helped improve adolescents’ lifelong satisfaction and receive more support from their teachers and peers. In Model II, trust in communication between caregivers and adolescents significantly affected adolescents’ positive emotions, while alienation caused conflicts between caregivers and adolescents.

There are several pieces of advice for adolescents from migrant families to improve their lifelong satisfaction. First, they could adopt a positive attitude towards family relations and communicate more with their parents or caregivers. Second, seek environmental support: Connect with peers who share similar experiences. Join community groups or organizations that provide support and understanding to adolescents from migrant families. Building a supportive network can help adolescents feel less isolated and more empowered. Additionally, adolescents could try to remember some activities that help improve positive affect and emotions when surrounded by negative emotions. Of course, it is also possible to ensure correlations among social cognitive variables. It is possible to enhance self-efficacy, goal progress, outcome expectations, and academic satisfaction to improve the lifelong satisfaction of migrant workers’ children by asking caregivers to cooperate with consultation; similarly, this can improve caregiver-child communication, trust, propel regulation, and coactivity. However, reducing caregiver-child alienation and conflicts can also improve the well-being of migrant children. Furthermore, this study revealed that good health conditions are essential to all social and cognitive factors. Parents and caregivers could enhance the frequency and quality of coactivity and communication with adolescents in their families. For example, they could cook together, engage in conversations on topics of interest to the adolescents, participate in sports activities, and demonstrate more trust in them.

Moreover, there are several directions for school mental health centres to improve their service and help students adjust their social cognitive factors and emotions properly. This study’s results demonstrate that mental health consultation and therapy services in many Chinese vocational schools do not meet students’ needs, especially in VET schools in rural areas. Additionally, the research findings offer valuable suggestions for increasing the well-being of Chinese migrant workers’ children and VET school students. School consultants should help students gather academic support from parents or caregivers, teachers, and fellow students. Moreover, external services ought to benefit from the connected advantages of combining interventions on self-efficacy, outcome expectations, and goal progress with those on environmental support. This outcome is achievable through integrating interventions related to students’ efficacy beliefs, expectations, and goal setting to increase students’ academic satisfaction and lifelong well-being. Additionally, more attention should be given to students with more negative affect, and more efforts should be made to improve their positive affect. For instance, practitioners should organize group activities and workshops to address negative emotions and affect and lead students to find the best way to release stress and sadness. Additionally, consultants can use social media, such as WeChat and QQ. to offer guidelines whenever and wherever the students need. Furthermore, students should be guided and encouraged to learn more about their personality traits and characteristics; cultivate more hobbies; and help them find their flow.

## Implications, limitations, and directions for future research

This paper initially involved family relations in the SCWB model. Although this study has promising results, there are still some limitations. First, this was a cross-panel study that recorded only the current information on migrant workers’ children in VET schools. Some students had left behind before and migrated in recent years or months, and some had lived with their parents before and were sent back to their hometown when they entered high school. Therefore, mental health is different at different time points. While this study is based on a cross-sectional survey, in future studies, long-term investigations could compare the differences among various periods.

Second, the participants were teenagers in China’s vocational high schools; many studies have shown that family migration impacts young children in primary school or early childhood. However, there are still limited studies that include the SCWB. Therefore, further studies could extend the family-related SCWB to migrant workers’ children in primary school and kindergarten.

Third, only the family-related SCWB was explored. Other interpersonal relationships are also essential for migrant workers’ children; for example, fellow and teacher-student relations are also valuable variables to examine in the SCWB model in a collectivist context. Further research could extend the social cognitive model to other interpersonal relation variables, such as the school relations SCWB.

The results indicate that the children of migrant workers can increase their lifelong satisfaction and self-efficacy by engaging in positive activities, such as sports, reading, sketching, singing, dancing, or playing musical instruments. Adopting a positive attitude regarding school achievement, which leads to improved academic performance, is critical to overall well-being. Furthermore, when experiencing unpleasant emotions, children should try to recall activities to boost their affect and emotions. Of course, connections between social cognitive characteristics could also be used. Increasing self-efficacy, goal progress, outcome expectations, and academic satisfaction among migrant workers’ children is achievable by allowing caregivers to participate in consultation to improve caregiver-child communication, trust, and motivation.

### Supplementary Information

Supplementary Information 1.

Supplementary Information 2.

## Data Availability

All data generated or analysed during this study are included in this published article [and its supplementary information files].

## References

[1] National Bureau of Statistics of the People’s Republic of China. *Seventh national population census bulletin (No. 7).* https://www.stats.gov.cn/sj/zxfb/202302/t20230203_1901087.html (2021).

[2] Yang Y (2024). Mobility or stability? The dilemma of mental health development among children of rural migrant workers in cities and consideration of countermeasures. J. Ment. Health Educ. Prim. Second. Sch..

[3] Wang B, Li S, Li GM, Du H (2024). Survey on the mental health status of rural left-behind children in the context of rural revitalization and the construction of a cloud service platform. J. Liaoning Educ..

[4] Lent RW (2004). Toward a unifying theoretical and practical perspective on well-being and psychosocial adjustment. J. Couns. Psychol..

[5] Işık E, Ulubey E, Kozan S (2018). An examination of the social cognitive model of well-being in Turkish college students. J. Vocat. Behav..

[6] Jia Z, Tian W (2010). Loneliness of left-behind children: a cross-sectional survey in a sample of rural China. Child Care Health Dev..

[7] Dai Q, Chu RX (2018). Anxiety, happiness and self-esteem of western Chinese left-behind children. Child Abuse Negl..

[8] Chen X, Liang N, Ostertag SF (2017). Victimization of children left behind in rural China. J. Res. Crime Delinq..

[9] Chen M, Chan KL (2016). Parental absence, child victimization, and psychological well-being in rural China. Child Abuse Negl..

[10] Liu LJ, Sun X, Zhang CL, Wang Y, Guo Q (2010). A survey in rural China of parent-absence through migrant working: The impact on their children's self-concept and loneliness. BMC Public Health.

[11] Lu Y, Yeung JW, Liu J, Treiman DJ (2019). Migration and children's psychosocial development in China: When and why migration matters. Soc. Sci. Res..

[12] Vocational Education Department (2008). Some Opinions of Ministry of Education on Further Deepening Education Reform of Secondary Vocational Education.

[13] Zhao C, Wang F, Li L, Zhou X, Hesketh T (2017). Long-term impacts of parental migration on Chinese children's psychosocial well-being: Mitigating and exacerbating factors. Soc. Psychiatry Psychiatr. Epidemiol..

[14] Jordan LP, Graham E (2012). Resilience and well-being among children of migrant parents in South-East Asia. Child Dev..

[15] Cortes P (2015). The feminization of international migration and its effects on the children left behind: Evidence from the Philippines. World Dev..

[16] Graham E, Jordan LP (2011). Migrant parents and the psychological well-being of left-behind children in Southeast Asia. J. Marriage Fam..

[17] Mazzucato V (2015). International parental migration and the psychological well-being of children in Ghana, Nigeria, and Angola. Soc. Sci. Med..

[18] Zhao K (2007). The epidemiological characteristics of unintentional injuries among children left-behind in Anhui Province. J. Dis. Control Prev..

[19] Chan A, Crothall EG (2009). Paying the Price for Economic Development: The Children of Migrant Workers in China.

[20] Goe R, Ipsen C, Bliss S (2018). Pilot testing a digital career literacy training for vocational rehabilitation professionals. Rehabil. Couns. Bull..

[21] China Youth Daily (2008). Expert Claims the Problem of Left-Behind Children is so Serious it Might Endanger the Future of Rural China.

[22] China Youth Research Centre (2006). China’s Children and Juveniles Statistical Handbook.

[23] Guang’an Municipal Party (2006). A Survey of the Leftbehind Children in Guang’an City, Sichuan Province.

[24] Gao L, Zheng X, Yan BB (2010). The differences of well-being between the east and the west: From the view of self-construal. Adv. Psychol. Sci..

[25] Valtolina GG, Colombo C (2012). Psychological well-being, family relations, and developmental issues of children left behind. Psychol. Rep..

[26] Ling M (2015). “Bad students go to vocational schools!”: Education, social reproduction and migrant youth in urban China. China J..

[27] Chai X, Li X, Ye Z, Li Y, Lin D (2019). Subjective well-being among left-behind children in rural China: The role of ecological assets and individual strength. Child Care Health Dev..

[28] Patton GC (2016). Our future: A lancet commission on adolescent health and wellbeing. Lancet.

[29] Moore GF (2018). School, peer and family relationships and adolescent substance use, subjective wellbeing and mental health symptoms in wales: A cross sectional study. Child Indic. Res..

[30] Wu, Z. H. & Li, J. M. The realistic dilemma and policy choice of migrant workers’ migrant children receiving compulsory education in cities. *Educ. Res.* (2016).

[31] Huang Y, Song Q, Tao R, Liang Z (2018). Migration, family arrangement, and children's health in China. Child Dev..

[32] Langton CE, Berger LM (2011). Family structure and adolescent physical health, behavior, and emotional well-being. Soc. Serv. Rev..

[33] Xiong, M. & Ye, Y. D. A review of the research on the mental health of the children of rural migrant workers in China. *Adv. Psychol. Sci.* 1798–1813 (2011).

[34] Lent RW, Brown SD, Hackett G (1994). Toward a unifying social cognitive theory of career and academic interest, choice, and performance. J. Vocat. Behav..

[35] Bandura A, Freeman WH, Lightsey R (1999). Self-efficacy: The exercise of control. J. Cogn. Psychother..

[36] Bandura A (1986). Social Foundations of Thought and Action: A Social Cognitive Theory.

[37] Fouad NA, Guillen A (2006). Outcome expectations: Looking to the past and potential future. J. Career Assess..

[38] Zhang FY (2014). From, "confucian well-being" to "gentleman well-being"-mencius' four directions of well-being. Qilu J..

[39] Lu L (2007). Well-being of Chinese people. Discov. Appl. Phsychol..

[40] Zhong J, Arnett JJ (2014). Conceptions of adulthood among migrant women workers in China. Int. J. Behav. Dev..

[41] Sheu HB, Chong SS, Chen HF, Lin WC (2014). Well-being of Taiwanese and Singaporean college students: Cross-cultural validity of a modified social cognitive model. J. Couns. Psychol..

[42] Christopher JC (1999). Situating psychological well-being: Exploring the cultural roots of its theory and research. J. Couns. Dev..

[43] Celeste G, Puritz P (2002). Children left behing: An assessment of access to counsel and quality of representation in delinquency proceedings in Louisiana. SUL Rev..

[44] Aneshensel CS, Sucoff CA (1996). The neighborhood context of adolescent mental health. J. Health Soc. Behav..

[45] Lent RW (2005). Social cognitive predictors of academic interests and goals in engineering: Utility for women and students at historically black universities. J. Couns. Psychol..

[46] Sheu HB, Liu Y, Li Y (2017). Well-being of college students in China: Testing a modified social cognitive model. J. Career Assess..

[47] Leiter MP, Schaufeli WB (1996). Consistency of the burnout construct across occupations. Anxiety Stress Cop..

[48] Lent RW, Brown SD (2006). Integrating person and situation perspectives on work satisfaction: A social-cognitive view. J. Vocat. Behav..

[49] Hu Q, Schaufeli WB (2009). The factorial validity of the Maslach Burnout inventory-student survey in China. Psychol. Rep..

[50] Armsden GC, Greenberg MT (1987). The inventory of parent and peer attachment: Individual differences and their relationship to psychological well-being in adolescence. J. Youth Adolesc..

[51] Central Government of the People’s Republic of China (2019).

[52] Xu T (2019). Perception on risk factors of child maltreatment in China: A qualitative study among health professionals. BMJ Open.

[53] Tur-Porcar A, Mestre V, Llorca A (2015). Parenting: Psychometric analysis of two studies in Spanish population. Anu. Psicol..

[54] Lee RM, Choe J, Kim G, Ngo V (2000). Construction of the Asian American family conflicts scale. J. Couns. Psychol..

[55] Sluzki CE (1979). Migration and family conflict. Fam. Process.

[56] Diener E (1996). Traits can be powerful, but are not enough: Lessons from subjective well-being. J. Res. Pers..

[57] Lyubomirsky S (2001). Why are some people happier than others? The role of cognitive and motivational processes in well-being. Am. Psychol..

[58] Veenhoven R, Michalos AC (2005). Is happiness a trait?. Citation Classics from Social Indicators Research: The Most Cited Articles Edited and Introduced by Alex C. Michalos.

[59] Tellegen A, Waller NG, Boyle GJ, Matthews G, Saklofske DH (2008). Exploring personality through test construction: Development of the multidimensional personality questionnaire. The SAGE Handbook of Personality Theory and Assessment.

[60] Lawrence JW, Carver CS, Scheier MF (2002). Velocity toward goal attainment in immediate experience as a determinant of affect. J. Appl. Soc. Psychol..

[61] Ryan RM, Deci EL (2001). On happiness and human potentials: A review of research on hedonic and eudaimonic well-being. Annu. Rev. Psychol..

[62] Sanderson CA, Cantor N, Cervone D, Shoda Y (1999). A life task perspective on personality coherence: Stability versus change in tasks, goals, strategies, and outcomes. The Coherence of Personality: Social-Cognitive Bases of Consistency, Variability, and Organization.

[63] DeNeve KM (1999). Happy as an extraverted clam? The role of personality for subjective well-being. Curr. Dir. Psychol. Sci..

[64] Heppner PP, Lee DG, Snyder CR, Lopez SJ (2002). Problem-solving appraisal and psychological adjustment. Handbook of Positive Psychology.

[65] Argyle M, Argyle M (2003). 18 causes and correlates of happiness. Well-being: The Foundations of Hedonic Psychology.

[66] National Bureau of Statistics of the People’s Republic of China. *2017 migrant workers monitoring survey report.* http://www.stats.gov.cn/tjsj/zxfb/201804/t20180427_1596389.html (2018).

[67] Meng X, Manning C, Meng X, Manning C (2010). The great migration in China and Indonesia: trends and institutions. The great migration.

[68] Ministry of Civil Affairs of People’s Republic of China. *Data of national left-behind children in 2018.* http://www.mca.gov.cn/article/gk/tjtb/201809/20180900010882.shtml (2018).

[69] Net DT (2005). Communique on the 2005 Education Development Plan; Understanding the Key Issues.

[70] Ezeofor I, Lent RW (2014). Social cognitive and self-construal predictors of well-being among African college students in the US. J. Vocat. Behav..

[71] Mundfrom, D. J. & Whitcomb, A. *Imputing missing values: the effect on the accuracy of classification.* https://eric.ed.gov/?id=ED419817 (1998).

[72] National Bureau of Statistics of the People’s Republic of China. *2018 migrant workers monitoring survey report.* http://www.stats.gov.cn/tJSj/zxfb/201904/t20190429_1662268.html (2019).

[73] Gibson JW (1992). Compensating for missing data in social work research. Soc. Work Res. Abstr..

[74] Diener E, Emmons RA, Larsen RJ, Griffin S (1985). The satisfaction with life scale. J. Pers. Assess..

[75] Pavot W, Diener E (2008). The satisfaction with life scale and the emerging construct of life satisfaction. J. Posit. Psychol..

[76] Sheu HB, Mejia A, Rigali-Oiler M, Primé DR, Chong SS (2016). Social cognitive predictors of academic and life satisfaction: Measurement and structural equivalence across three racial/ethnic groups. J. Couns. Psychol..

[77] Lent RW (2011). Predicting the job and life satisfaction of Italian teachers: Test of a social cognitive model. J. Vocat. Behav..

[78] Duffy RD, Lent RW (2009). Test of a social cognitive model of work satisfaction in teachers. J. Vocat. Behav..

[79] Watson D, Clark LA, Tellegen A (1988). Development and validation of brief measures of positive and negative affect: The PANAS scales. J. Pers. Soc. Psychol..

[80] Fischer JA (2009). Subjective well-being as welfare measure: Concepts and methodology. Munich Pers. RePEc Arch..

[81] Tomșa R, Jenaro C (2015). Children left behind in romania: Anxiety and predictor variables. Psychol. Rep..

[82] Currie C (2008). Researching health inequalities in adolescents: The development of the health behaviour in school-aged children (HBSC) family affluence scale. Soc. Sci. Med..

[83] Hu Q, Bentler PM (1999). Cutoff criteria for fit indexes in covariance structure analysis: Conventional criteria versus new alternatives. Struct. Equ. Model. Multidiscip. J..

[84] Xu L, Cheung M, Leung P, Xu Y (2018). Migrant child phenomenon in China: Subjective happiness factors for assessing service needs. Child. Youth Serv. Rev..

