# Supplementary material for: Family migration and well-being of Chinese migrant workers’ children
Source: Sci Rep. 2024 Jun 4;14:12862. doi: 10.1038/s41598-024-63589-5 (PMC11150384; doi:10.1038/s41598-024-63589-5)
Supplement: Supplementary file 1 — Supplementary Information 1. [file 41598_2024_63589_MOESM1_ESM.pdf]

A technical appendix for the SEM modelling

1.Bief Introduction of SEM

Structural equation modelling (SEM) is a diverse set of methods used by scientists doing both observational and experimental research (Bollen, Kenneth A, 1989). It involves a model representing how various aspects of some phenomenon are thought to causally connect to one another (Bollen, Kenneth A, 1989), moreover, containing postulated causal connections among some latent variables (variables thought to exist but which can't be directly observed). Regarding the testing, the commonly used fit statistics include Chi-square, Akaike information criterion (AIC), Root Mean Square Error of Approximation (RMSEA), Comparative Fit Index (CFI), etc. (Tarka, Piotr , 2017).

2. SEM in our study

Structural equation modeling in AMOS 22 was used to test the structural validity of these models within the target population. The bootstrap method has been used to test indirect effects related to environmental support, self-efficacy, and caregiver-child coactivity; 2,000 bootstrap samples were generated to estimate the indirect effects and bias-corrected 90% confidential intervals. Here, three fit indices were used to assess the data fit: CFI (the comparative-fit-index), RMSEA (the root mean squared error of approximation), and root mean squared residual (SRMR). Values of RMSEA $\leq$ 0.08, CFI $\geq$ 0.90, and SRMR $\leq$ 0.08 are considered an adequate fit, and RMSEA $\leq$ 0.06, CFI $\geq$ 0.95, and SRMR $\leq$ 0.05 are considered a good fit (Hu & Bentler, 1999). Additionally, researchers have determined that models meeting only one of the three values (CFI, RMSEA, and SRMR) can be considered to have acceptable fit [38]. The results of confirmatory factor analysis (CFA) of the above model with the current data set show an acceptable fit, with x^2= 8829, df =1621, p < 0.0001, CFI = 0.846, RMSEA = 0.051 and SRMR = 0.076.

Positive affect and caregiver-child coactivity have indirect effects on lifelong satisfaction, the indirect (mediated) effect of caregiver-child coactivity on the positive affect is 0.114 and with statistically significant (P < 0.001). Moreover, environmental support and self-efficacy have significant indirect effects on outcome expectation; self-efficacy and outcome expectation have significant indirect effects on academic satisfaction. The significant standard indirect effects for lifelong satisfaction ranged from -0.287 to 0.283, whereas those for academic satisfaction ranged from 0.100 to 0.310.

Figure 1 shows the research hypothesis that most paths are with statistical significance. Moreover, most of the paths between SCWB variables are strongly correlated with each other, for example, the paths' coefficient from environmental support to self-efficacy, self-efficacy to goal progress, goal progress to academic satisfaction and academic satisfaction to lifelong satisfaction are both above 0.4. Furthermore, the family relationship variables are closely correlated with each other; the paths' coefficient from caregiver-child coactivity to communication frequency is above 0.4, communication frequency to regulation is above 0.2 and caregiver-child coactivity to regulation/house rules is above 0.1. Most paths are statistically significant as it was predicted. This finding is consistent with the results of other studies [2,38]. Moreover, the path from communication frequency to environmental support is bigger than 0.2, which is easy to understand as students with better communication with caregiver(s) or parents receive more support from their caregiver(s)/parents. What is unexpected is that the path from goal progress to academic satisfaction is not significant.

Figure 1: Family Relationship and Social Cognitive Model I -Path Coefficients

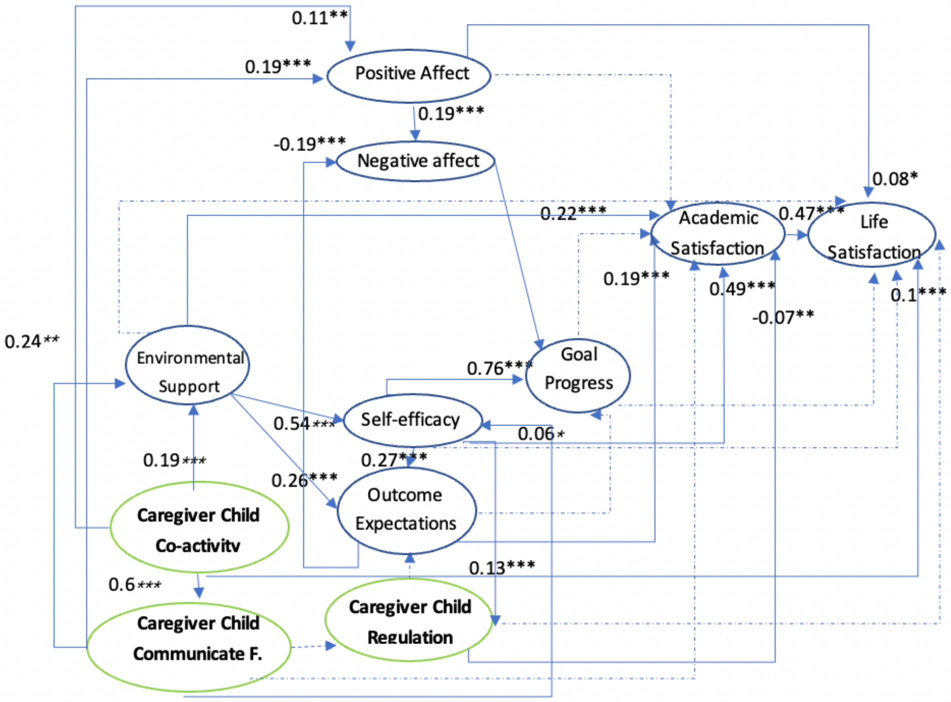

Note: Communicate. F. is the short form for Caregiver-child communication frequency in figure 1.

The results in Figure 1 show the coefficients, covariance, and p-value of all paths in the research hypothesis, which are with statistical significance, except for nine paths ($P > 0.05$). The path coefficients for many social cognitive variables (from environmental support to self-efficacy, from self-efficacy to goal progress, from environmental support to outcome expectation, from goal progress to academic satisfaction, and from academic satisfaction to lifelong satisfaction) showed similar results with previous studies [38] related to the SCWB model. Most of the path coefficients with statistical significance were above zero, except the path coefficient of the path from outcome expectation to negative affect ($path\ coeficient = -0.19, P < 0.001$). The path coefficient of the path from self-efficacy to goal progress was the largest ($path\ coeficient = 0.76, P < 0.001$), which means that when the self-efficacy improves 1 unit, the goal progress increases 0.76 units and *ceteris paribus*.

Figure 2 : Family Relationship and Social Cognitive Model II -Path Coefficients

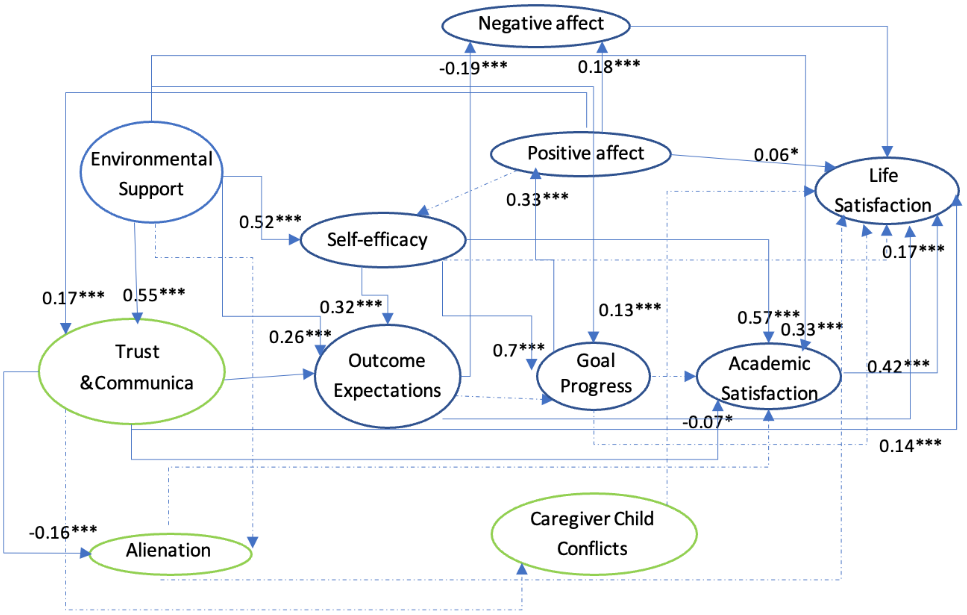

Note: Trust & communica. is short for Caregiver-child trust & communication, Alienation is short for Caregiver-child alienations, conflict is short for Caregiver-child conflicts in this figure 2.

This is followed by the path from caregiver-child coactivity to caregiver-child communication frequency ($path\ coeficient = 0.60, P < 0.001$), the path from environmental support to self-efficacy ($path\ coeficient = 0.54, P < 0.001$), the path from self-efficacy to academic satisfaction ($path\ coeficient = 0.49, P < 0.001$), and the path from academic satisfaction to lifelong satisfaction ($path\ coeficient = 0.47, P < 0.001$). Meanwhile, it is not significant for the path from caregiver-child coactivity to caregiver-child regulation, the path from caregiver-child regulation to outcome expectation, and another nine paths. Furthermore, many paths with statistical significance are between caregiver-child relationship variables and SCWB variables, for example, the path from caregiver-child coactivity to positive affect ($path\ coeficient = 011, P < 0.001$), the path from self-efficacy to caregiver-child regulation ($path\ coeficient = 0.13, P < 0.001$), the path from caregiver-child communication frequency to positive affect ($path\ coeficient = 0.19, P < 0.001$), and the path from caregiver-child coactivity to lifelong satisfaction ($path\ coeficient = 0.11, P < 0.001$). It implies that more communication and co-activities between a child and caregiver(s) can improve the quality of the self-efficacy, positive affect, and lifelong satisfaction of Chinese VET school students. Caregiver-child regulation was negatively correlated with academic satisfaction but positively correlated with outcome expectation.

Figure 2 shows the research hypothesis of the second family relationship SCWB model, which included two of the caregiver-child attachment variables (caregiver-child communication & trust and alienation) and caregiver-child conflict. The paths between social cognitive well-being variables were based on previous SCWB studies [37,38]. The paths between SCWB variables and family relationship variables were based on the characteristics of Chinese VET school students from migrant families. In the hypothesis model, self-efficacy, outcome expectation, goal progress, and academic satisfaction bridged the correlation paths between environmental support and lifelong satisfaction.

The structural equation modeling analysis results for the child-caregiver attachment and conflict social cognitive model with the current data shows an acceptable fit also, with x^2= 7576.2, df = 1740, p < 0.0001, CFI = 0.861, RMR = 0.068 and RMSEA = 0.045. The coefficient of four significant paths is above 0.5 (shown in figure 6), which are the path from environmental support to caregiver-child trust & communication, the path from environmental support to self-efficacy, the path from self-efficacy to

goal progress, and the path from self-efficacy to academic satisfaction. Moreover, coefficients of the other four paths are above 0.3, which includes the path from environmental support to academic satisfaction, the path from self-efficacy to outcome expectation, the path from academic satisfaction to lifelong satisfaction, and the path from alienation to conflicts. Moreover, four of the significant paths are below 0; that means the correlation between these two variables is negative, for example, the path from environmental support to alienation, the path from outcome expectation to negative affect, the path from trust & communication to alienation and the path from alienation to lifelong satisfaction. Furthermore, the coefficient of other paths in this model is above 0.

References

Bollen, Kenneth A. (1989). Structural equations with latent variables. New York: Wiley. ISBN 0-471-01171-1. OCLC 18834634.

Principles and practice of structural equation modeling (4th ed.). New York. ISBN 978-1-4625-2334-4. OCLC 934184322.

Tarka, Piotr (2017). "An overview of structural equation modeling: Its beginnings, historical development, usefulness and controversies in the social sciences". Quality & Quantity. 52 (1): 313–54. doi:10.1007/s11135-017-0469-8. PMC 5794813. PMID 29416184.
